# Forebrain nuclei linked to woodpecker territorial drum displays mirror those that enable vocal learning in songbirds

**DOI:** 10.1371/journal.pbio.3001751

**Published:** 2022-09-20

**Authors:** Eric R. Schuppe, Lindsey Cantin, Mukta Chakraborty, Matthew T. Biegler, Electra R. Jarvis, Chun-Chun Chen, Erina Hara, Mads F. Bertelsen, Christopher C. Witt, Erich D. Jarvis, Matthew J. Fuxjager

**Affiliations:** 1 Department of Biology, Wake Forest University, Winston-Salem, North Carolina, United States of America; 2 Department of Neurobiology and Behavior, Cornell University, Ithaca, New York, United States of America; 3 Laboratory of Neurogenetics of Language, The Rockefeller University, New York, New York, United States of America; 4 Department of Neurobiology, Duke University Medical Center, Durham, North Carolina, United States of America; 5 Howard Hughes Medical Institute, Chevy Chase, Maryland, United States of America; 6 Center for Zoo and Wild Animal Health, Copenhagen Zoo, Frederiksberg, Denmark; 7 Museum of Southwestern Biology and Department of Biology, University of New Mexico, Albuquerque, New Mexico, United States of America; 8 Department of Ecology, Evolution and Organismal Biology, Brown University, Providence, Rhode Island, United States of America; University of Zürich, SWITZERLAND

## Abstract

Vocal learning is thought to have evolved in 3 orders of birds (songbirds, parrots, and hummingbirds), with each showing similar brain regions that have comparable gene expression specializations relative to the surrounding forebrain motor circuitry. Here, we searched for signatures of these same gene expression specializations in previously uncharacterized brains of 7 assumed vocal non-learning bird lineages across the early branches of the avian family tree. Our findings using a conserved marker for the song system found little evidence of specializations in these taxa, except for woodpeckers. Instead, woodpeckers possessed forebrain regions that were anatomically similar to the pallial song nuclei of vocal learning birds. Field studies of free-living downy woodpeckers revealed that these brain nuclei showed increased expression of immediate early genes (IEGs) when males produce their iconic drum displays, the elaborate bill-hammering behavior that individuals use to compete for territories, much like birdsong. However, these specialized areas did not show increased IEG expression with vocalization or flight. We further confirmed that other woodpecker species contain these brain nuclei, suggesting that these brain regions are a common feature of the woodpecker brain. We therefore hypothesize that ancient forebrain nuclei for refined motor control may have given rise to not only the song control systems of vocal learning birds, but also the drumming system of woodpeckers.

## Abstract 2

Vocal learning is thought to have evolved in three orders of birds (songbirds, parrots, and hummingbirds). This study shows that woodpeckers have evolved a set of brain nuclei to mediate their drum displays, and these regions closely mirror those that underlie song learning in songbirds.

## Introduction

Advanced vocal learning is a rare trait, which thus far has been found in only 3 of over 40 avian lineages (songbirds, parrots, and hummingbirds) and 5 of over 30 mammalian lineages (humans, cetaceans, pinnipeds, elephants, and bats; [1]). Among primates, humans are the only vocal learner, where this trait is a critical component of spoken language. Similar to their behavior, vocal learning birds and humans have been found to have specialized forebrain circuits for the acquisition and production of learned vocalizations [2]. These vocal learning brain regions are embedded in or adjacent to ancient forebrain motor pathways, and they show specialized expression of specific genes [3–5]. Vocal nonlearning birds, including close relatives to vocal learners, have thus far been found to either not contain these forebrain regions or contain rudimentary regions without the same gene expression specializations [4–6]. For these reasons, it is proposed that vocal learning pathways might have arisen by the duplication of brain pathways from surrounding motor learning circuits, which subsequently become specialized for sets of genes involved in neural connectivity, physiology, and plasticity [2,4,7]. This idea also suggests that similar circuits might have evolved for other specialized communication behaviors, such as those that demand extraordinary performance skill and exquisite motor control.

**Fig 1:**
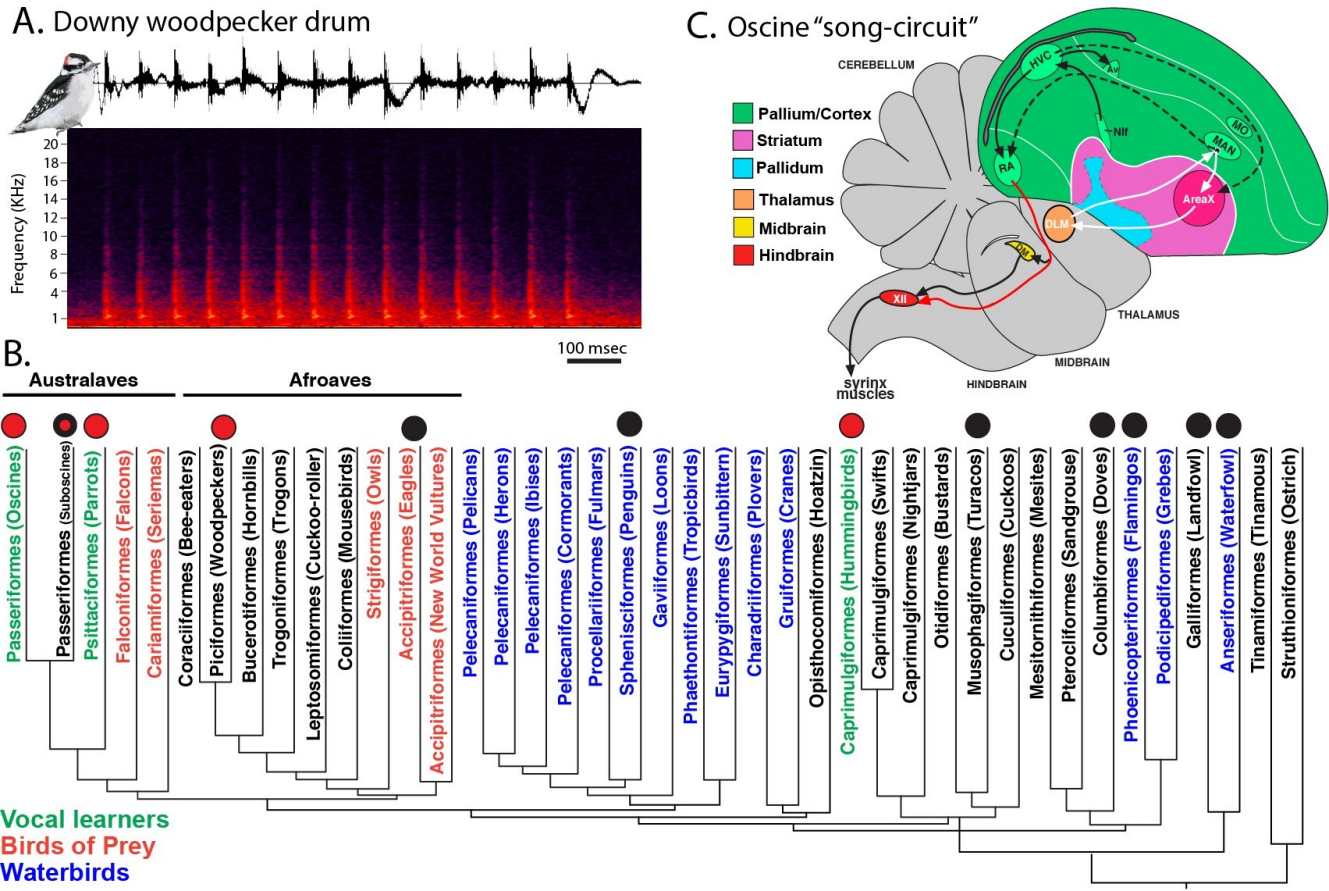
Brain regions identified for vocalization and drumming behavior across the avian phylogeny. (A) Waveform and spectrogram diagrams of a spontaneous male downy woodpecker drum recorded at dawn during the breeding season. Image of downy woodpecker from Greg Schechter (CC Public Domain via WikiMedia). (B) Avian phylogeny from [73] further annotated where specialized brain regions have been either identified [red circles: oscine songbirds, parrots, hummingbirds; red circle with black outline: suboscines, which only has RA-like nucleus), and woodpeckers] or investigated and not found (black circles). Note that this figure includes the 7 genera never examined before our current study. (C) Schematic diagram of the oscine “song circuit” that is characterized by a set specialized forebrain song nuclei essential for producing learned vocalizations. This circuit is composed of 2 main interconnected neural pathways: the vocal motor pathway (VMP; HVC and RA) and anterior forebrain pathway (AFP; LMAN, Area X, and DLM). While the VMP is necessary for both the acquisition and production of learned song, the AFP largely only necessary for acquisition.

Woodpecker drumming is a highly specialized communication behavior that is performed when individuals rapidly hammer their bill on a tree to generate specific patterns of sounds. Drumming is used to help negotiate territorial interactions (**Fig 1A**), much like birdsong in some species [8–10], and thus it is markedly different than other woodpecker beak behaviors, such as drilling for food and excavating nest cavities in old trees [9]. Field studies show that increasing drum speeds (beats/s), or increasing drum length by a few beats, profoundly enhances the display’s threat to competitors [8,10]. In fact, birds will attempt to match the tempo of these high-speed drums, even if many individuals fall short of this feat [8,9]. Additionally, a drum’s rhythm encodes information about species identity, such that changes to its cadence and/or acceleration distort the signal’s recognizability to conspecifics [8,10,11]. Studies in multiple woodpecker species also suggest that drums may encode individual identity [12–14] and that woodpeckers can distinguish drums produced by their neighbors compared to those that they have never encountered before [14]. Furthermore, similar to oscine and suboscine [15] passerine birds, woodpeckers appear to have a protracted drum ontogeny, in which signal production becomes less variable during their second breeding season [14]. Thus, content-based selection on drumming should strongly favor the evolution of mechanisms that mediate exquisite motor control of the head and neck to generate an optimal communication signal. Although it is unclear whether woodpecker drumming has a learned component, one possibility is that the mechanisms at the level of the brain may resemble those that mediate vocal communication in vocal learning birds (**Fig 1B and 1C**).

Here, we screened the brains of 7 key avian species that represent major lineages assumed to consists of only vocal nonlearners but that have never been tested for such specialized brain regions (**Fig 1B and 1C**). We used the *parvalbumin* (*PV*) gene, which is highly conserved and constitutively up-regulated in the adult forebrain song nuclei of all avian vocal learners examined to-date and the speech cortical regions of humans, but not in their closest vocal nonlearning relatives [4,16,17]. PV codes for a Ca^2+^ buffering protein, involved in neuroprotection and neural plasticity [18,19]. We found no clear evidence of analogous telencephalic brain nuclei with specialized *PV* expression in the nonvocal learning birds examined, with one notable exception—the woodpeckers (**Fig 1B**). These birds contained several pallial nuclei with specialized *PV* up-regulation, which were in regions where song nuclei of vocal learners are located. However, instead of showing activation during vocalization, these regions showed activation during drumming. Our findings suggest that the drumming system of woodpeckers and the song system of vocal learning birds may have evolved by parallel neural mechanisms.

### Phylogenetic screening for forebrain display nuclei

We obtained fresh frozen brains of species representing major branches of land and water bird clades (**Fig 1B**). These included samples representing several Neoaves clades: (i) Harris hawk (*Parabuteo unicinctus*) and downy woodpecker (*Dryobates pubescens*) in the Afroaves clade; (ii) Humboldt penguin (*Spheniscus humboldti*) in the core waterbird clade; (iii) red-crested turaco (*Tauraco erythrolophus*) in the Otidimorphae clade, which is sister to the Caprimulgimorphae and thus includes vocal learning hummingbirds; (iv) American flamingo (*Phoenicopterus ruber*) in the Phoenicopterimorphae clade, which is sister to all other Neoaves (including all vocal learners, representing the deepest branch of Neoaves we assessed); (v) domestic duck (*Anas platyrhynchos domesticus*) in the Anseriformes clade, which is sister to Galliformes (and therefore includes previously tested vocal nonlearning chickens and quails [4,16]); and (vi) emu (*Dromaius novaehollandiae*) in the Palaeognathae clade, which represents the deepest branch of all living avian species. In addition, we included male zebra finches (*Taeniopygia castanotis*) [4,16], a vocal learning oscine songbird species that is in the Australaves clade and sister to the Afroaves (which includes woodpeckers). Nine parrot species in Australaves were reported on separately [17], but the sections were processed at the same time using identical probes and methods. We focused on males in all species, given that males always retain vocal learning behavior and the associated brain regions whenever sex differences exist. We serially sectioned one brain hemisphere sagittally and the other coronally. We hybridized approximately 50 to 200 serial sections spaced ≥100 μm (depending on brain size) with RNA probes for *PV* expression. The up-regulation of *PV* in specific pallial song/speech control regions has been found thus far in all 3 vocal learning bird lineages and humans, but not within pallial subdivisions thus far in any vocal nonlearning species, such as quail, chickens (Galliformes), ring doves (Columbimorphae), mice, and marmosets (among nonhuman primates) [4,16,17,20,21]. The intensity of specialized *PV* expression levels in vocal learning pallial regions are among the highest within the pallium, on par with the intercalated nidopallium primary sensory regions (auditory Field L2, visual Entopallium, and somatosensory Basorostralis). We also processed adjacent sections for gene expression of the transcription factor *FoxP1*, which has a pattern that unambiguously allows determination of avian forebrain subdivision boundaries, more so than the standard Nissl staining [22].

**Fig 2:**
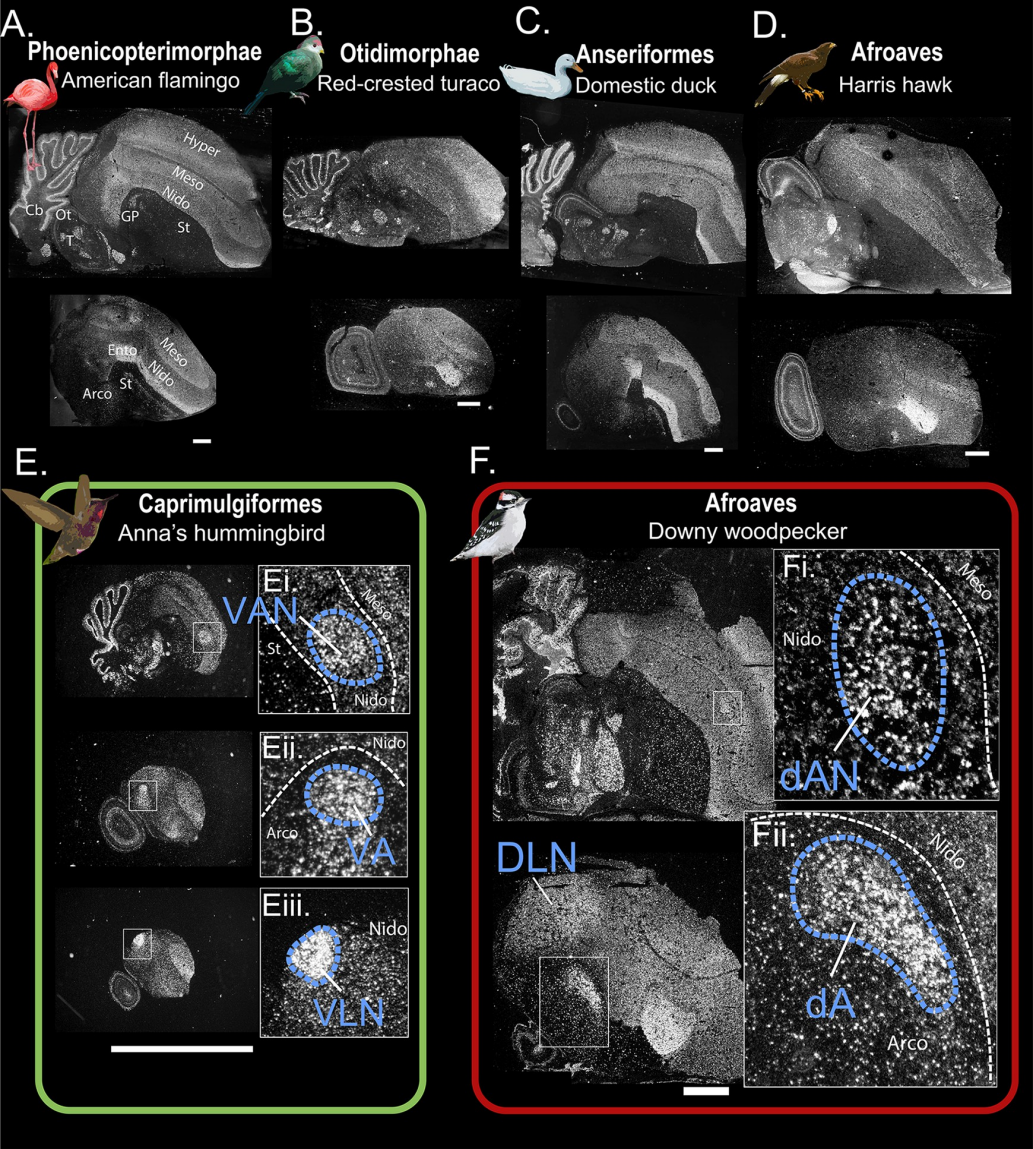
Identification of forebrain structures with specialized *parvalbumin* (*PV*) mRNA expression. (A-F) Representative radioactive in situ hybridization microscope images of *PV* mRNA in species representing 8 different avian orders (see S1 Fig for penguin and emu). *PV*-rich forebrain nuclei were present in only (E) vocal learning hummingbirds (positive control) and (F) downy woodpeckers. (Ei-iii) High magnification of 3 telencephalic “song control” nuclei in hummingbirds. (Fi) High magnification of the woodpecker drumming nucleus of the anterior nidopallium (dAN); and (Fii) drumming nucleus of arcopallium (dA). Each scale bar is equal to 2 mm. Neuroanatomical markers shown in “A” are as follows: Hyper, hyperpallium; Meso, mesopallium; Nido, nidopallium; GP, globus pallidus; T, Thalamus; Ot, optic tectum; St, striatum; Arco, arcopallium; Ento, entopallium. White dashed lines in high-magnification images (Ei-iii and Fi-ii) indicate boundaries for different telencephalic regions (e.g., boundary between nidopallium and mesopallium), whereas blue dashed lines indicate specialized PV regions identified in Anna’s hummingbirds and downy woodpeckers. *Image credits*: *flamingo from Wilfredo Rodríguez; turaco from Edelmauswaldgeist; duck from Orso della campagna e Papera dello stagno; hawk from Cheva; hummingbird from Stickpen*, *and downy woodpecker from Greg Schechter*. *All image licenses*: *CC Public Domain via WikiMedia*.

We found broadly similar patterns of *PV* gene ecxpession in telencephalic subdivisions across all species, with highest expression in primary sensory input cell populations (intercalated nidopallium, intercalated hyperpallium) and the pallidum. We found intermediate levels of *PV* within the secondary regions (nidopallium and hyperpallium) and tertiary regions (mesopallium), and low levels of *PV* in the striatum, but with sparse highly labeled cells (**Figs 2A–2D, S1A and S1B**). These patterns helped reveal differences in the shapes of some brain subdivisions among species, consistent with the known diversity of avian brain organization [23]. Aside from these pallial subdivisions, we identified *PV* in Purkinje cells, thalamic nuclei, globus pallidus in all species (**Figs 2, S1 and S2 and S1 Table**). Together, this suggests that this *PV* probe hybridized well across all species.

**Fig 3:**
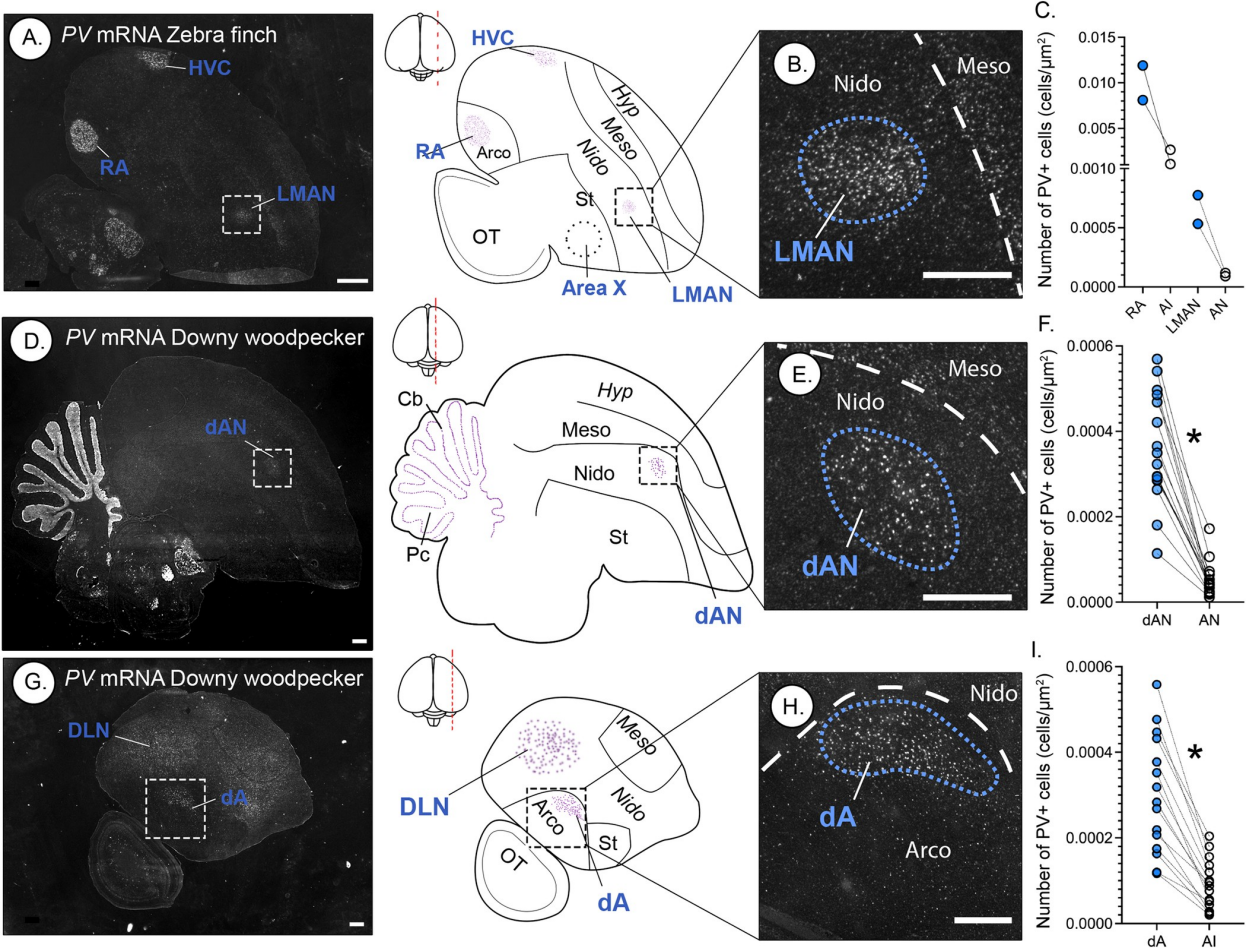
*Parvalbumin* (*PV*) specializations in the forebrain of male zebra finches and male downy woodpeckers. (A) Representative microscope image from inverted black and white colormetric in situ hybridization at low magnification of zebra finch brain sections, showing specialized up-regulation of *PV* mRNA expression in 3 of the 7 telencephalic song control nuclei including RA, HVC, and LMAN. Schematic diagrams next to the microscope image illustrate key neural anatomical subdivisions. Dashed circle in the zebra finch schematic drawing is the relative location of Area X as determined by other molecular markers on adjacent sections (see S4 Fig). (B) High-magnification image of the zebra finch LMAN area. (C) Number of *PV*+ cells in 2 song control nuclei (RA and LMAN) compared to the expression in the surrounding arcopallium (intermediate arcopallium [AI]) or nidopallium (anterior nidopallium [AN]) in adult male zebra finches (*n* = 2). (D-I) Representative images from low (D, G) and high (E, H) magnification with schematic illustrations of neuroanatomical regions with PV (dA, DLN, and dAN). (F, I) Graphical illustrations of the significant up-regulation of *PV* in the male downy woodpecker in (F) dAN (*n* = 16; *t*_*30*_ = 9.06, *p* < 0.001) and (I) dA (*n* = 16, *t*_*30*_ = 5.91, *p* < 0.001) relative to the surrounding forebrain regions (AN and AI, respectively). Hyp, hyperpallium; arco, arcopallium; nido, nidopallium; Ot, optic tectum; St, striatum; Cb, cerebellum; Pc, cerebellar Purkinje cells. Scale bars represent 500 μm. Dashed lines in high-magnification images indicate boundaries for different telencephalic regions (e.g., boundary between nidopallium and mesopallium), whereas blue dashed lines indicate specialized *PV* regions identified in zebra finches and downy woodpeckers. Asterisk (*) denotes significant differences between putative drumming nucleus and the surrounding pallium (*p* < 0.05). Data for C, F, and I can be found in S1 Data.

Next, we confirmed the specialized upregulation of *PV* in the song nuclei of male Anna’s hummingbird (*Calypte anna*) (**Fig 2E**), zebra finches (a songbird) (**Fig 3A–3C**), and 9 parrot species [17] (**S2A Fig**). Similar to previous findings in quails and doves [4,16], we did not find clear evidence of such specialized regions. This included locations where one would expect to see song nuclei in the song learning birds, such as the intermediate arcopallium, caudal and anterior nidopallium in central to far lateral planes, or adjacent ventral mesopallium. Further, we found no up-regulated *PV* expression in other putative nuclei within these same brain subdivisions, nor in other telencephalic subdivisions (e.g., hyperpallium, dorsal mesopallium, striatum, and pallidum). While our sample size for the nonvocal learning species was limited (*n* = 1 individual per species), our data across species are consistent with the idea that birds such as the hawk, turaco, flamingo, penguin, emu, and duck do not have neural song control nuclei with elevated levels of *PV* expression. Nonetheless, it is possible that other markers might show rudimentary vocal substrates, as in suboscines [15].

### A specialized set of nuclei specific to woodpeckers

We found one striking exception to the results described above, which was the downy woodpecker (**Figs 2F and 3D–3I**). Males of this species showed 3 regions of specialized up-regulated *PV* expression in or near areas where one would expect to find pallial song nuclei, particularly in songbirds and hummingbirds (**Figs 2E and 3A–3C**). We gave these brain regions different names than in vocal learning birds, keeping with the tradition of calling potentially convergent areas by different names [24–26]. The first woodpecker region was within the anterior nidopallium (**Figs 2Fi, 3D–3F, S2B and S3**), but slightly dorsal to where one would expect to find the songbird lateral magnocellular nucleus of the anterior nidopallium (LMAN; **Fig 3A–3C**), the hummingbird vocal nucleus of the anterior nidopallium (VAN; **Fig 2Ei**), and the parrot oval nucleus of the anterior nidopallium (NAO; **S2A Fig**), all of which are involved in song learning and modulation [2]. In woodpeckers, we called this region the putative drumming nucleus of the anterior nidopallium (dAN). Both songbird LMAN and woodpecker dAN exist at the boundary of the nidopallium and mesopallium (**Fig 3A–3C and 3D–3F**). Interestingly, the songbird LMAN is round in shape (**Fig 3B**), whereas the woodpecker dAN is oval (**Figs 2Fi, 3D, and 3E**), more like the hummingbird VAN (**Fig 2Ei**) and parrot NAO (**S2A Fig**) analogs. In songbirds and downy woodpeckers, *PV* expression in LMAN and dAN is approximately 5× greater than the surrounding nidopallium (anterior nidopallium [AN]; **Fig 3A–3F**).

The second region was in the intermediate arcopallium (**Figs 2Fii and 3G–3I**). This area began medially on the dorsal surface of the medial arcopallium, where one would expect to find the hummingbird vocal nucleus of the arcopallium (VA) analog (**Fig 2Eii**), and ended laterally more centrally to where one would expect to find the songbird robust nucleus of the arcopallium (RA) analog involved in song production (**Figs 3A, S4A and S4B**). We called this woodpecker region the putative drumming nucleus of the arcopallium (dA). Note that while this region exhibits substantially higher *PV* compared to the surrounding arcopallium (**Fig 3G–3I**), the PV expression is not as dense as in songbirds (see **Fig 3A–3C**). We further confirmed its presence in the arcopallium with hybridization in adjacent sections to arcopallium specific markers [27] *Lim homeobox 9 (Lhx9)* and *ETS translocation variant 1 (ETV1)* (**S4C, S5A, and S5B Figs**).

The third woodpecker region was a large area within the dorsal lateral nidopallium (DLN; **Figs 2F, 3G, 3H, S3C and S3D**). This area had a similar shape and relative location in the nidopallium (below the lateral ventricle and hippocampus) to where one can find both the hummingbird vocal nucleus of the lateral nidopallium (VLN; **Fig 2Eiii**) and the songbird HVC (**Fig 3A**). However, *PV* expression in this part of the woodpecker DLN appeared much larger in relative size than in these 2 other species (**Figs 3G,** S3C and S3D**)** and thus was more comparable to that of the large *PV*-enriched NLC complex analog for vocal production in parrots (which is positioned more laterally in the nidopallium (**S2A Fig**) [17]). Importantly, we did not identify such *PV* specializations in the lateral nidopallium of the other species that we examined (**Figs 2A–2D and S1**), much like in previous examinations of vocal nonlearners [4,16]. Thus, it is possible that this DLN region of the woodpecker brain is comparable to the HVC analogs in vocal learning avian species.

With the exception of the parrot mesopallium oval nucleus (MO), specialized *PV* expression is not found in mesopallial song nuclei across vocal learning birds [16,17]. Accordingly, we did not find *PV* specializations in the mesopallium dorsal to dAN, Field L2 or basorostralis, or anywhere else in the mesopallium. Further, unlike the song nuclei in the 3 vocal learning lineages, we could not find prominent Nissl staining differences in morphology in the regions with specialized *PV* expression in the downy woodpecker. Conversely, after discovering the *PV* specializations in the downy woodpecker, we repeated our scan in the other vocal nonlearning species brains where one might expect to find such nuclei, but we did not see anything. The combined results indicate that the downy woodpecker has regions with *PV* specializations in the arcopallium and nidopallium that closely resembles those in vocal learning species, in particular songbirds and hummingbirds, but with some important differences.

### *Parvalbumin* (*PV*) specializations occur in both sexes and other woodpecker species

**Fig 4:**
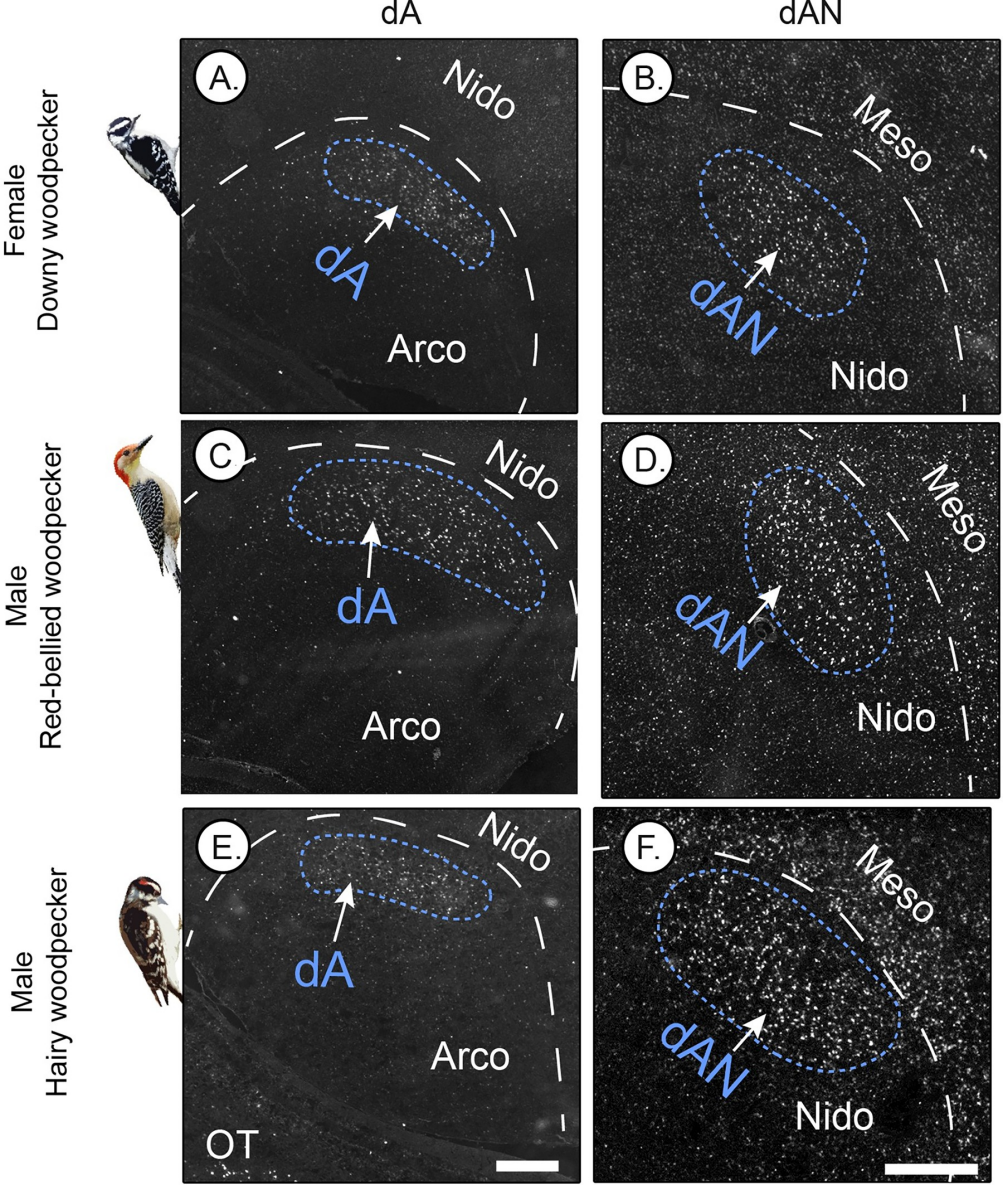
*Parvalbumin* (*PV*) specialization in dorsal arcopallial (dA) and nidopallial (dAN) nuclei in female downy woodpeckers, as well as 2 other woodpecker species. (A, B) Female downy woodpecker. (C, D) Male red-bellied woodpecker. (E, F) Male hairy woodpecker. Shown are representative in situ hybridization microscope images (inverted black and white colormetric) of *PV* expression (white), with fast red as a counterstain (grey). Nido, nidopallium; Meso, mesopallium; Arco, arcopallium; Ot, optic tectum. Scale bars = 500 μm. White dashed lines in images indicate boundaries for different telencephalic regions. White dashed lines in high-magnification images indicate boundaries for different telencephalic regions (e.g., boundary between nidopallium and mesopallium), whereas blue dashed lines indicate specialized *PV* regions identified in woodpeckers. *Photo credits*: *Female downy woodpecker from Ken Thomas (Public Domain via WikiMedia); male red-bellied woodpecker from Neal Lewis (Public Domain Mark 1*.*0 via Flickr); and male hairy woodpecker from David Whelan (Public Domain via WikiMedia)*.

Both male and female downy woodpeckers drum [10]. Thus, we would expect to find the aforementioned brain regions in both sexes, if these areas were in fact involved in drumming behavior. We obtained 3 female downy woodpeckers, and we found the same regions of specialized up-regulated *PV* expression in dAN, dA, and DLN (**Figs 4A, 4B, S3E and S3F**).

Nearly all extant woodpecker species drum to mediate sociosexual interactions [28], and thus we tested whether other taxa in the woodpecker family show the same *PV* specializations as the downy woodpecker. We examined the brains of male hairy woodpeckers (*Leuconotopicus villosus*, *n* = 2) and red-bellied woodpeckers (*Melanerpes carolinus*, *n* = 3). Although both taxa are distantly related to downy woodpeckers, they showed *PV* specializations similar forebrain nuclei as in male downy woodpeckers (**Fig 4C–4F**).

### Lack of detectable specialization thus far in the woodpecker striatum

Because *PV* does not show convergent specialized expression in striatal vocal regions, we sought another marker that does exhibit convergent expression patterns in the striatal vocal nucleus of vocal learners (including humans [4])—regulator of G-protein signaling 12 (*RGS12*). Specialized up-regulation of this gene marks songbird Area X within the striatum, a nucleus involved in song learning, and the anterior vocal part of the human putamen within the striatum, which is involved in speech learning [4,29]. Whereas we confirmed a clear *RGS12* signature in zebra finch Area X (**S5C and S5E Fig**), we did not find such a signature in the striatum of the downy woodpecker (**S5D and S5F Fig**). Accordingly, an Area X–like nucleus in the woodpecker brain either does not exist or it does not share a common molecular signature of *RGS12* with vocal learning species.

### Specialized *PV* regions show correlated activation with territorial drumming behavior

We hypothesized that these regions either (i) were involved in a previously undiscovered vocal learning trait in woodpeckers; or (ii) corresponded to another complex motor behavior, such as drumming. Such a functional role would coincide with regional behaviorally regulated expression of immediate early genes (IEGs). To test these ideas, we staged simulated territorial intrusions (STIs) with playbacks of drumming to free-living resident downy woodpeckers for at least 30 min early in the morning when resident males normally produce dawn drums, or in the late afternoon when drum activity shows a subsequent peak [10,14,30]. We caught these individuals after the encounter and measured IEG expression in their brains using in situ hybridization. Residents performed varying amounts of drumming, agonistic vocalizations (whinny calls), and flight behavior to defend their territories. While many downy woodpecker residents produce innate whinny calls during territorial interactions [8,10], these vocalizations are produced at substantially lower rates compared to drums [14]. Many birds in the current study similarly produced relatively few whinny calls during the STI (mean: 1.77; range: 0 to 8 calls). However, these same birds produced approximately 10× more drums during the same period (mean 13.6: range: 0 to 57), providing further evidence that drums are the primary means of signaling during these agonistic interactions [9].

To assess whether IEG expression in specific parts of the brain correlate to variation in the production these different behaviors, we classified an individual’s behavioral responsivity to the same stimulus in one of 3 ways: (i) drummed (drummed only or drummed and called; see Materials and methods for more details); (ii) only called to playback; or (iii) neither drummed, nor called to playback. We also included a fourth group of downy woodpeckers that were not subject to an STI, but instead were passively caught (no playback) during the same times of the day that we otherwise staged encounters (such birds produced few, if any, calls or drums). We measured 2 IEGs: early growth response gene 1 (*EGR1*; also called *ZENK* in birds) and activity regulated cytoskeleton associated protein (*Arc*). Their transcript syntheses are up-regulated in response to neural activity in subsets of pallial and striatal neurons [15,25,26,31]. We focused specifically on IEG expression in dAN and dA, given that these regions were relatively more clearly defined through *PV* expression and that we lacked sufficient brain sections from the DLN. While we find clear drumming induced *EGR1* expression in dA (**S6A–S6F and S6M Fig**), woodpeckers lack *EGR1* in dAN regardless of behavioral condition (**S6G–S6L and S6N Fig)**. These findings are similar to the suboscine RA-like forebrain nucleus, which expresses *Arc* while vocalizing but not *EGR1*. Such anatomical differences in the induction of IEGs can be due to a host of factors, such as differences in cell type or magnitude of the transcriptional responses (IEG induction) following the protracted period of behavioral activity [32,33]. We do not expect that the lack of *EGR1* expression in the dAN is due to the latter of these possibilities, given that work in songbirds suggests that both *Arc* and *EGR1* exhibit similar expression patters following behavioral activation [31]. Since we see limited *EGR1* expression in the downy woodpecker, we focus on *Arc* expression to further investigate putative forebrain substrates that might related to drumming.

**Fig 5:**
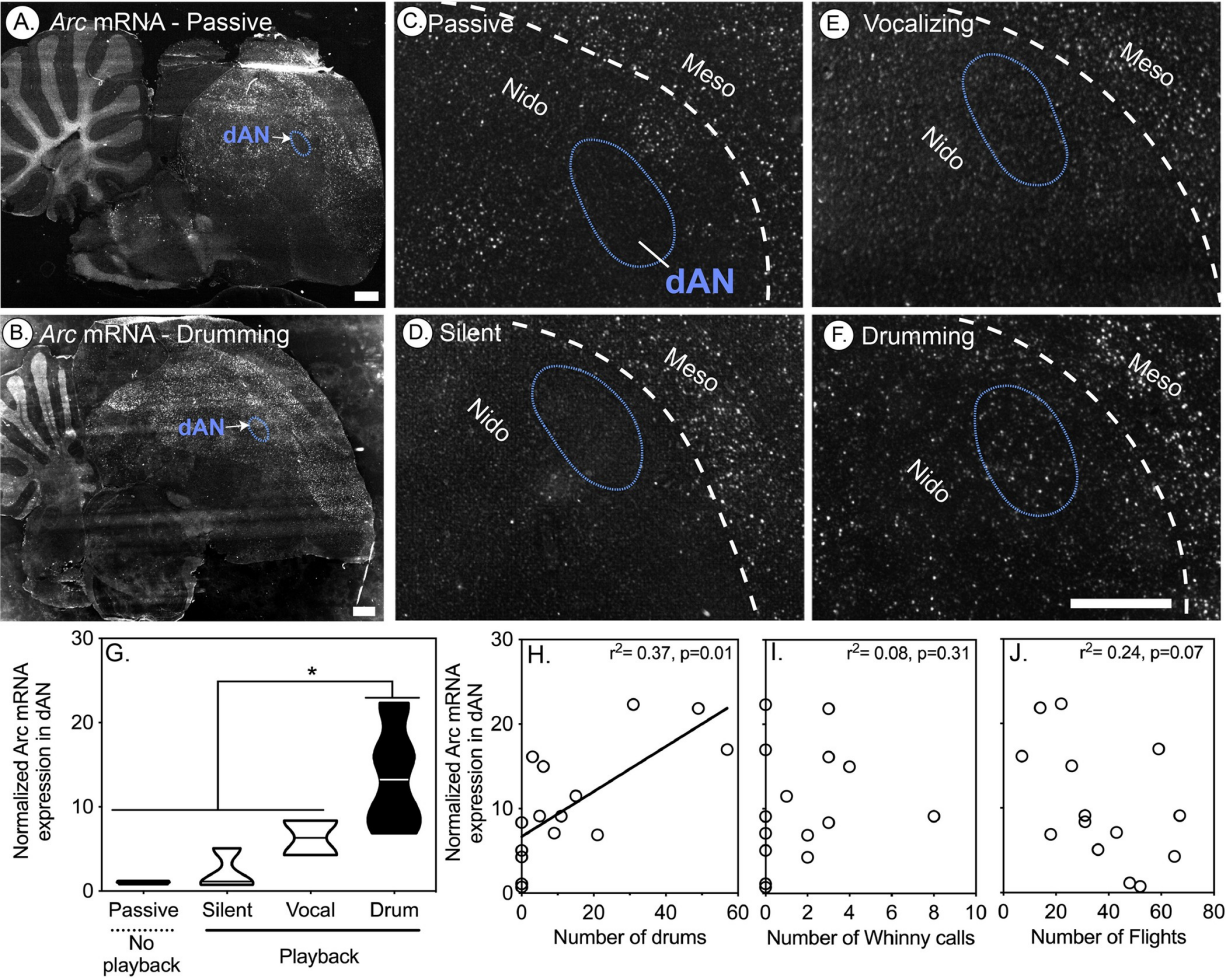
Activity induced *Arc* expression in the dorsal anterior nidopallium (dAN) of downy woodpeckers positively correlates with drumming behavior. (A-B) Representative low-magnification in situ hybridization microscope images of *Arc* expression (white), with fast red as a counterstain (grey) in downy woodpeckers that were either passively caught (A) or (B) after drumming during a simulated territorial encounter (STI). (C-F) Representative high-magnification examples of *Arc* mRNA image in (C) passively caught birds (*n* = 3) or birds that listened to STI playback (see Materials and methods) but either (D) did not produce vocalizations or drums (silent, *n* = 3), (E) produced only vocalizations and no drums (*n* = 2), or (F) produced drums (*n* = 10). Dash blue outline represents the *PV*-rich dAN area on each section determined from *PV* mRNA on an adjacent section. (G) Violin plots (horizontal line denotes median) illustrating differences in *Arc* gene expression in the *PV*-rich dAN in male downy woodpeckers caught after producing different behaviors. *Arc* mRNA expression significantly differed in the dAN (*F*_*3*,*14*_ = 21.14, *p* < 0.001), with drumming birds showing higher *Arc* expression than all other groups (all relevant comparisons: *p* < 0.01). (H) *Arc* mRNA expression in the dAN nucleus was positively correlated with the number of drums (*p* = 0.01). (I) No correlation was detected with the total number of aggressive vocalizations. Yet, (J) there was a marginally significant negative correlation with woodpeckers that flew less having greater *Arc* mRNA expression in dAN. Significant correlations denoted by solid lines (*p* < 0.05). Dashed white lines in images indicate boundaries between nidopallium and mesopallium, whereas blue dashed lines indicate specialized *PV* regions identified in downy woodpeckers. Data for G-J can be found in S2 Data.

**Fig 6:**
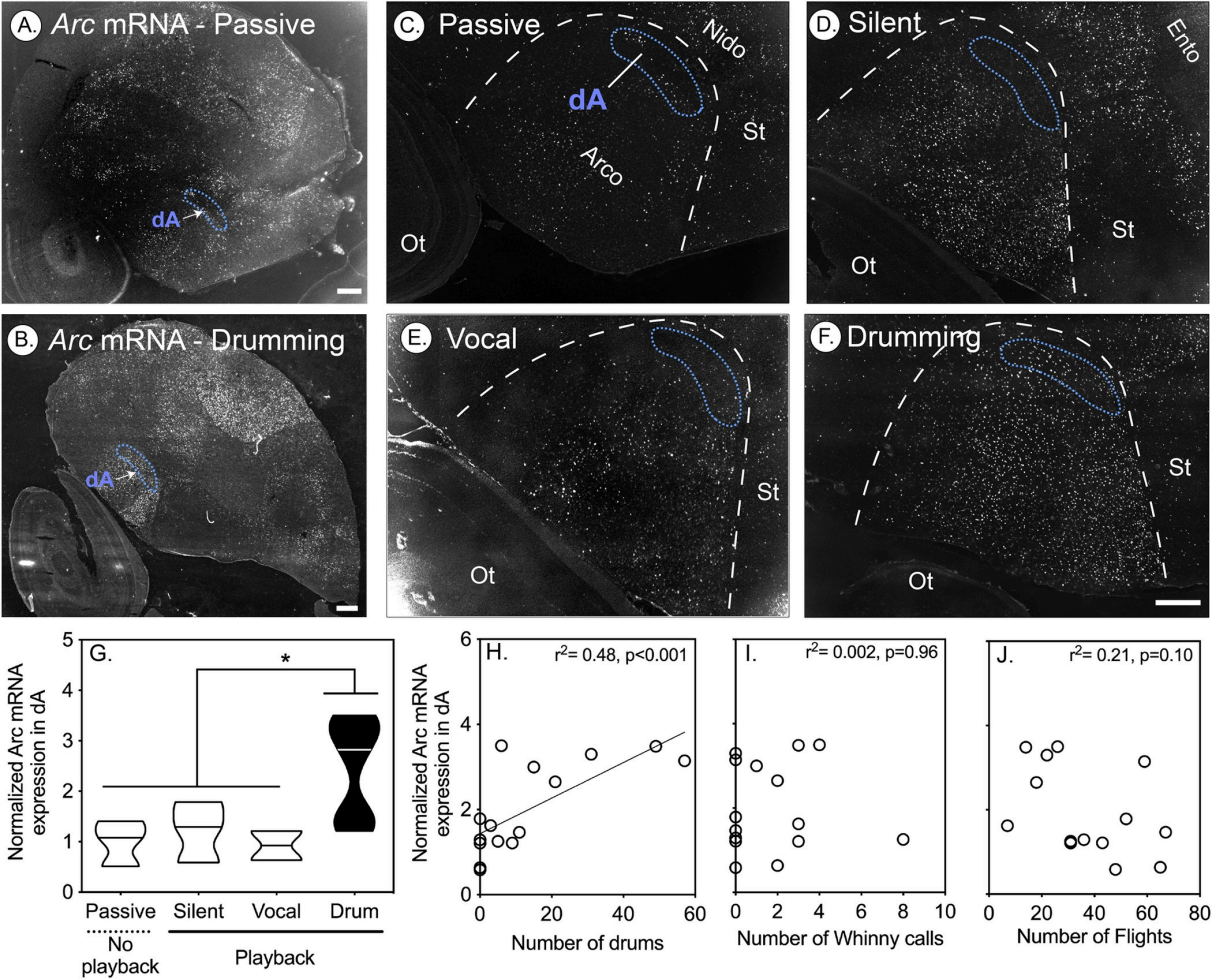
Activity induced *Arc* expression in the dorsal nucleus of the arcopallium (dA) of male downy woodpeckers positively correlates with drumming behavior. (A-B) Representative low-magnification in situ hybridization microscope images of *Arc* mRNA expression (white), with fast red as a counterstain (grey) in downy woodpeckers that were either passively caught (A) or (B) after drumming during a simulated territorial encounter (STI). (C-F) Representative high-magnification examples of *Arc* mRNA image in (C) passively caught birds (*n* = 3) or birds that listened to STI playback (see Materials and methods) but either (D) did not produce vocalizations or drums (silent, *n* = 3), (E) produced only vocalizations and no drums (*n* = 2), or (F) produced drums (*n* = 10). White dashed lines in high-magnification images indicate boundaries between the arcopallium and nidopallium, whereas blue dashed lines indicate specialized *PV* regions identified in downy woodpeckers. *PV*-rich dA area on each section was determined from *PV* mRNA on an adjacent slide. (G) Violin plots (horizontal line denotes median) illustrating in *Arc* gene expression in the dA of downy woodpeckers caught after producing different behaviors. IEG expression significantly differed in the dA (*F*_*3*,*14*_ = 4.74, *p* = 0.02), with drumming birds having significantly greater *Arc* mRNA compared to all other groups (all relevant comparisons: *p* < 0.05). (H) *Arc* mRNA expression in dA was positively correlated with the number of drums (*p* < 0.001). We did not find this relationship with *Arc* expression and the total number of aggressive vocalizations (I) or flights (J). Significant correlations have solid lines (*p* < 0.05). Data for G-J can be found in S2 Data.

Compared to passive controls, *Arc* was significantly up-regulated in the *PV*-rich dAN and dA nuclei in residents that responded to STI treatments by drumming, but not in those that responded only by vocalizing or by being silent (**Figs 5A–5G and 6A–6G**). In vocal learning birds, the amount of vocalizing-driven gene expression is correlated with the amount of song bouts produced in a 30-min window [34]. Similarly, in dAN and dA, we found a positive correlation between the number of neurons with *Arc* expression and the number of drums an individual produced (**Figs 5H and 6H**). No correlation was found for either the number of whinny vocalizations or the number of flights made (**Figs 5G, 5I, 5J, 6G, 6I, and 6J**). While there appeared to be a marginal negative correlation between flight and *Arc* mRNA expression in dAN, this effect was not significant (**Fig 5J**). These findings are therefore consistent with the idea that flights do not activate dAN, which further implies that both the dAN and dA are specifically associated with drumming behavior.

**Fig 7:**
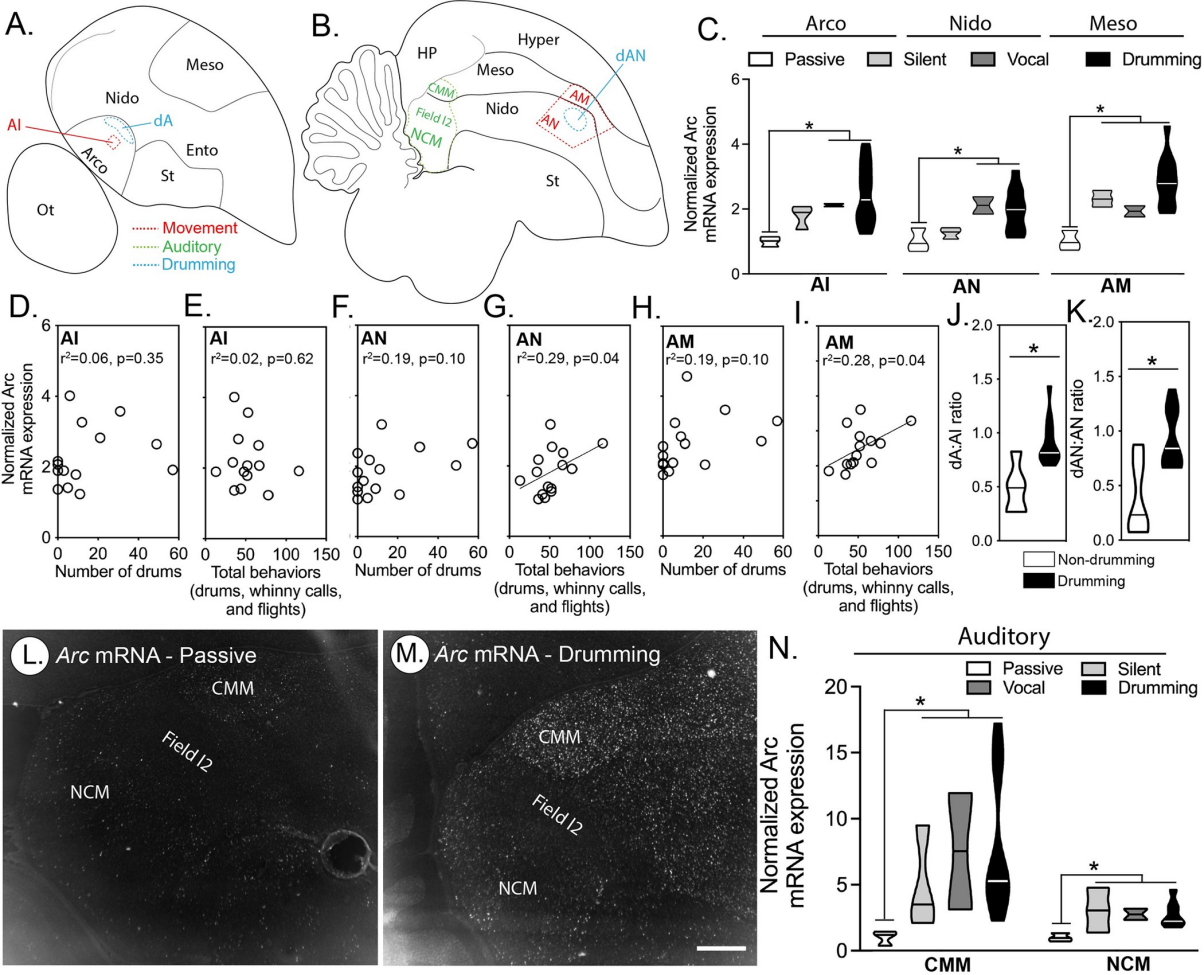
Movement and hearing-induced IEG expression in telencephalic regions of woodpeckers. (A-B) Schematic diagrams of sagittal brain sections for (A) lateral and (B) medial brain nuclei. (C) Violin plots (horizontal line denotes group median) illustrating significant differences in *Arc* mRNA expression in 3 movement related brain regions (AI: *F*_*3*,*14*_ = 3.99, *p* = 0.03, AN: *F*_*3*,*14*_ = 3.83, *p* = 0.03, AM: *F*_*3*,*14*_ = 10.50, *p* < 0.001) in male downy woodpeckers caught after hearing playbacks of drumming during simulated territorial intrusions (STIs) and either being silent in response or producing different behaviors. (D-I) No correlation was detected with the total number of drums and *Arc* expression in the (D) AI, (F) AN, or (H) AM (all *p*-values > 0.05). No correlation was detected between the total number of measured behaviors (drums, whinny calls, and flights) produced during the STI and *Arc* expression in the (E) AI (*p* > 0.05), but significant positive correlations were detected in the (G) AN and (I) AM (both *p*-values <0.05). Significant correlations have solid lines (*p* < 0.05). (J and K) Violin plots (horizontal line denotes group median) illustrating the ratio of *Arc* mRNA in putative drumming nuclei (e.g., dA (t_13_ = 3.10, *p* < 0.01) or dAN (t_13_ = 3.73, *p* < 0.01)) relative to the surrounding brain region (i.e., (J) AI or (K) AN, respectively). (L and M) Representative in situ hybridization microscope images of *Arc* expression (white) with fast red as a counterstain (grey) in a downy woodpecker that was (L) passively caught or (m) a bird that drummed while listening to a drumming playback during the STI. (N) Violin plots (horizontal line denotes group median) illustrating significant differences in *Arc* mRNA expression in 2 auditory forebrain nuclei (CMM: *F*_*3*,*13*_ = 4.91, *p* = 0.02; NCM: *F*_*3*,*13*_ = 4.05, *p* = 0.03) of downy woodpeckers caught after hearing playbacks of drumming and either being silent in response or producing different behaviors. Data for C-K and N can be found in S2 Data.

We also examined *Arc* expression around both the dAN and dA to further investigate whether these 2 nuclei are related specifically to drumming, as opposed to more general motor control. As such, we looked at the anterior nidopallium (AN), which is adjacent to dAN, and the intermediate arcopallium (AI), which is adjacent to dA (**Fig 7A and 7B**). Both the AN and AI are involved in motor control, and they express IEGs during production of body movements, such as wing flapping and hopping [3,25]. Relative to passively caught birds, *Arc* was higher in both the vocalizing and drumming individuals (**Fig 7C**); however, *Arc* in these 2 groups was statistically indistinguishable from birds that remained silent during an STIs (**Fig 7C**). While, we found no correlation between the number of flights individuals made during an STI and *Arc* expression in the AN (r^2^ = 0.01, *p* = 0.79) or the AI (r^2^ = 0.23, *p* = 0.07), although this latter effect showed a trend toward significance. Most importantly, drumming behavior produced during the STI was associated with neither *Arc* expression in the AN, nor the AI (**Fig 7D and 7F**). However, when we summed all movement related behaviors recorded (drums, whinny calls, and flights), we found a positive correlation with *Arc* expression in AN (**Fig 7G**), but not the AI (**Fig 7E**).

We also looked at *Arc* expression in the anterior mesopallium (AM), which is dorsal to AN (Fig 7B). The AM surrounds a small song nuclei (MO) and has also been implicated in movement [3]. Expression of *Arc* in this brain region was significantly elevated in all birds responding to STI (**Fig 7C**). Moreover, expression of *Arc* in the AM was also positively correlated with total behaviors exhibited during the STI (**Fig 7I**), but not significantly correlated with drumming itself (**Fig 7H**). In a final analysis, we investigated whether *Arc* expression in putative drum nuclei (e.g., dA and dAN) relative to the adjacent movement control regions (AI or AN) differed between drumming and nondrumming birds that were presented with playback. We found that *Arc* expression in both dA and dAN relative to the adjacent regions (AI and AN, respectively) was significantly greater in drumming birds, where it reached up to 50% greater compared to the surrounding tissue (**Fig 7J and 7K**)

### Drumming auditory stimulus and the auditory forebrain

As with birdsong, drumming and whinny calls in woodpeckers are acoustic communication signals [9,10]. As such, we might expect to see hearing-induced IEG expression in higher auditory areas of the woodpecker brain in the residents presented with an STI [3,35]. We therefore measured *Arc* expression in 2 key brain regions that underlie acoustic perception and auditory processing and that are known to have a hearing-induced IEG response [25,31]: the caudomedial nidopallium (NCM) and caudomedial ventral mesopallium (CMM). Relative to passive controls that did not hear drumming playback, all 3 groups that heard drumming in STIs showed induced Arc expression in NCM and CMM (**Fig 7L–7N**). Expression levels were similar regardless of whether listening birds performed drums, vocalizations, or remained silent (**Fig 7N**). These findings indicate that drumming sounds are a potent auditory stimulus for higher auditory regions of the woodpecker brain. Importantly, these findings are consistent with IEG activity in NCM in both lab and field studies of birds listening to playback [25,34].

## Discussion

**Fig 8:**
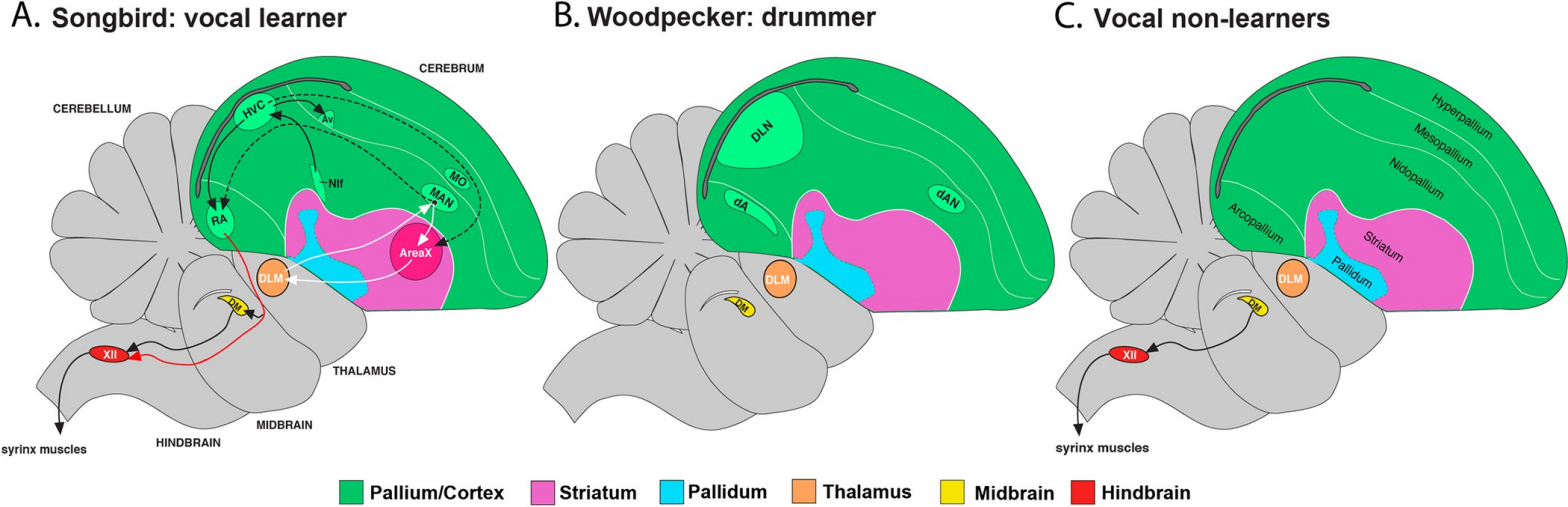
Schematic representation of putative drum control nuclei compared to similar nuclei in avian vocal learners. (A) Diagram showing the song nuclei (RA, HVC, LMAN, and Area X) and their connections (see Fig 1 and text for more details). (B) To date, a conserved molecular marker for drumming nuclei identified 3 telencephalic nuclei (dA, dNA, and DLN) of downy woodpeckers. (C) Nuclei for song learning and woodpecker drumming are absent in vocal nonlearns (chickens, quails, etc.).

In searching for brain regions underlying complex motor behaviors over a diverse range of bird lineages outside of well-known vocal learners, we identified a set of forebrain nuclei in woodpeckers that are reminiscent of song control nuclei in songbirds **(Fig 8)**. These regions include dA, dAN, and possibly DLN, which bear anatomical and molecular resemblance to the songbird RA, LMAN, and HVC, respectively (**Fig 8A and 8B**). Notably, these brain regions are not found in other avian lineages and thus appear to be specific to woodpeckers. However, we also show that, instead of controlling song, these specialized nuclei appear to be associated elaborate drumming behavior. This is a form of gestural communication that is unique to woodpeckers, and it is used to mediate conspecific territorial disputes (much like song is used in songbirds). Accordingly, the woodpecker dA and dAN showed drumming-driven IEG expression that was akin to the song nuclei’s singing-driven IEG expression. Moreover, these drumming-associated regions, again like the song nuclei of vocal learners, are adjacent to known motor regions in birds. These findings are, to our knowledge, the first to uncover the neural correlates of drumming in woodpeckers and more broadly nonvocal gestural signaling behavior outside of humans and their immediate primate ancestors.

### Specialized substrates for drum control

How might these putative forebrain nuclei influence woodpecker drumming behavior? We hypothesize that both regions play similar roles in drumming as the analogous regions play in regulating song in adult songbirds. For the dA, this may suggest that it is involved in premotor control of muscles that actuates movement programs for drumming [36]. The songbird RA modulates motoneurons that project to the syringeal muscles and the respiratory system [37]. We predict that the dA may therefore regulate downstream midbrain or hindbrain nuclei that regulate the motor output of the muscles of the head and neck, which actuate drum performance. Other research supports the possibility that the specialized premotor nuclei embedded within the song system, including those that mirror those found in male vocal learning birds, can control gesture. For instance, female songbirds perform copulation solicitation displays, and elements of the avian song system have been implicated in the control of display [38–40]. Although the core song system (e.g., HVC-RA) projections remain intact in females, hindbrain projecting neurons have been seemingly modified to possibly allow for modulation of reproductive displays (see [41]).

For the dAN, according to known functions of the songbird LMAN [42], we hypothesize that this region may help regulate context-specific changes in drum structure. In songbirds, LMAN and RA are differentially activated in undirected song, compared to female directed contexts [43–45]. In general, variability in LMAN activity contributes to variability in RA neurons during singing, allowing for spectral features of song to become more variable [46]. Conversely, inactivation or lesions of LMAN in adult animals can not only reduce acoustic variability, but also prohibit plastic changes in adult song [46]. Aside from these changes, there is also evidence that LMAN modulates song tempo and that LMAN firing is temporally patterned ([44,45,47,48]; but see [49,50] that show the role of HVC in regulating tempo). The latter finding suggests that LMAN might modulate RA activity to adjust song at specific points in time. These findings in LMAN lead us to hypothesize that dA, and possibly DLN, may control how woodpeckers rapidly adjust the speed of their drums during territorial interactions [8]. This includes flexibly adjusting the speed of their own drums in an attempt to match the speed of territorial intruders [8]. These characteristics of the signal are critical to its behavioral function, encoding important aspects of species identity and threat level of the signaler [8,10,28,51]. If dAN shares functional similarities to the song circuit, we would expect that this nidopallial nucleus in woodpecker exhibits connections with the arcopallium. Indeed, evidence in songbirds suggests that LMAN and shell have parallel input to RA and dorsal arcopallium, respectively [52].

The lack of specialization in either mesopallium (e.g., MO) or striatal (e.g., Area X) song counterparts for drumming behavior in woodpeckers does not mean these regions do not exist. A putative woodpecker MO or Area X may not have the specialized expression of genes (e.g., *PV*, *RGS12*, or *FoxP1*) that we examined thus far, or these 2 areas may be difficult to identify with IEGs (particularly when surrounded by motor brain regions active with other movements that occur within the same time frame). To test whether other such nuclei exist, future experiments will need to examine additional genes that mark song nuclei and/or influence the woodpecker’s ability to perform drums. Furthermore, investigating IEG activity following spontaneous dawn drumming might reveal more nuclei and greater differential IEG expression. Previous studies on songbirds, for example, suggest that song nuclei exhibit more variable singing-driven IEG expression in the field. [53]. Importantly, there was much greater variation in Area X in the birds that responded with singing to playbacks than in other song nuclei. [53]. Furthermore, younger songbirds seem to have higher *Arc* expression when passively singing in the morning [54]. Thus, performing experiments in which woodpeckers spontaneously drum in a laboratory setting may yield similar results. Of course, it is also possible that a specialized region within the striatum does not exist in woodpeckers. This would suggest that, woodpeckers only require a limited number of forebrain nuclei to refine and produce their displays [15]. Support for this for idea comes from developmental studies suggesting that LMAN is involved in subsong production and forms functional synapses with (and thus communicates to) RA prior to HVC [55–57]; yet, without HVC, these birds fail to crystalize on a normal adult song [57]. If woodpeckers only require limited sensorimotor exploration to produce species specific drums as adults, then the nuclei in woodpeckers may be sufficient to generate these displays.

While our current IEG work provides clear evidence that brain regions like dA and dAN are likely activated during drumming, one important caveat to point out is that we delineate boundaries of the 2 focal nuclei for IEG quantification using *PV* expression from adjacent sections (approximately 12 μm apart). This practice is common and has been employed in multiple vocal learning species [3,58] and in suboscines [15]; yet, in theory, it may lead to a slight under- or overestimation of the number of *Arc* expressing cells. At the same time, evidence suggests such tracing through adjacent sections would not alter our findings. Namely, PV is densely expressed in both the dA and dAN but almost completely absent in the surrounding arco- and nidopallium, respectively. *Arc*, for example, is roughly 3- to 10-fold higher in the dA and dAN of drumming birds compared to passively caught birds. Moreover, even though passive and silent birds have *Arc* in the brain surrounding that surround dA and dAN, this transcript is conspicuously absent in these 2 putative drum nuclei. In fact, we find that overall IEG expression in putative drum nuclei relative to the surrounding pallium is comparable to field collected hummingbirds and suboscines [3,15]. Future research on neurophysiological recordings and inactivation of these brain areas will help resolve these questions, though such work is can be quite challenging with wild-caught birds [25].

### Functional implications

One of the major implications from our findings is that woodpeckers might learn components of their species-typical drum. This notion is based on the simple fact that in songbirds, RA, HVC, and LMAN play an important role not only in song production, but song learning, especially during early ontogeny [46]. Yet, it is currently unclear what aspects of drumming might be acquired through learning. One possibility is that all aspects of drumming are learned early in life, much like in song learning birds. By contrast, another possibility is that individuals learn specific features of the drum in a way that modifies an existing innate motor template for the behavior. Species-specific rhythm, for example, might be acquired through learning [59,60]; it varies among woodpecker taxa and encodes species identity [51]. Even slight changes to a species’ rhythm—regardless of overall drum speed or length—can profoundly affect the signal’s content [8,51], which means that selection likely favors a motor mechanism that helps enforce this characteristic of the signal. Similarly, even subtle individual differences in the speed or rhythm of drumming can alter its effect during territorial interactions. Studies that explore the learned basis of drumming are underway.

Consistent with the second hypothesis above is emerging work in songbirds and suboscines that suggests that nuclei within the song circuit can coordinate and modify acoustic features of unlearned vocalizations [15,61,62]. In suboscine birds, for example, an RA-like nucleus is essential for mediating precise motor commands for song output in reproductively active adults, as well as the motor refinement of vocalizations during a protracted song ontogeny period early in life [15]. To this end, lesions in this RA-like area in a suboscine noticeably impacts vocal performance [15], with the pitch and fundamental frequency of their songs becoming substantially more variable. Many acoustic features of drums become more stable in a downy woodpecker’s second breeding season compared to their first [14]. This suggests, like some suboscines [15,63], that woodpeckers potentially have a protracted drum ontogeny wherein they practice these signals, leading to enhanced display performance later in life.

### Evolution of putative drum control nuclei

Our current results are consistent with the idea that selection for woodpecker drumming occurs through the simultaneous exaptation of an ancient forebrain motor pathway that evolved originally to help regulate facets of avian display behavior. Existing models of brain circuit evolution [2,64] suggest that neural circuits established for one purpose can become evolutionarily reconfigured for another purpose [65]. One might therefore expect that rudimentary forebrain substrates that govern display behavior appeared early in the avian phylogeny and were then subject to “evolutionary tinkering” as a way to support display diversification. If so, then it is possible that anatomically and molecularly conserved neural substrates across woodpeckers and vocal learning birds have similar functions (see above). Like songbirds, woodpeckers must not only produce drums at temporally precise species-specific rhythms [8,51,66], but also have the flexibility to refine motor output based on context [8].

Another nonmutually exclusive possibility is that specialized brain areas for drumming evolved from antecedent motor learning systems. The antecedent pathway may consist of segments from the arcopallium, nidopallium, and mesopallium, which otherwise control body and limb movement [2]. Thus, putative drum control nuclei may have emerged as a specialized region within existing motor pathways that control complex movements, such as those that govern head and neck motion. Initial support for this idea comes from our findings that show regions that surround dA and dAN exhibit greater Arc expression in birds that were active, compared to passively caught animals. Future tracing work is underway to more fully address this idea. For either hypothesis, they would suggest that the song system of vocal learning birds and the drumming system of woodpeckers had common origins.

## Conclusions

Diverse species incorporate different types of body movements into their communication repertoires, and in many cases, these signals evolve to extremes [67,68]. How the brain has evolved to support the production and refinement of these signals throughout times is unclear. Here, we identify several forebrain nuclei that appear analogous to the cortical substrates within the song circuit of vocal learning birds. We expect that these substrates arose from an ancient motor pathway around them. Other gestural displays across the avian phylogeny may utilize similar neural circuitry to coordinate and refine elaborate body and limb movements.

## Materials and methods

### Animals

To screen for the presence or absence of song nuclei with specialized gene expression across species, we obtained brains of 11 species (plus 9 previously analyses parrot species as positive controls): (i) passively caught adult male and female downy woodpeckers (*Dryobates pubescens*) collected in both Winston-Salem, North Carolina (male *n* = 4, female *n* = 3) and Albuquerque, New Mexico (male: *n* = 2, female: *n* = 1); (ii) passively caught adult male red-bellied woodpeckers (*Melanerpes carolinus*; *n* = 3) collected in Winston-Salem, North Carolina; (iii) passively caught adult male hairy woodpeckers (*Leuconotopicus villosus*; *n* = 2) collected in Winston-Salem, North Carolina; (iv) a male Harris hawk (*Parabuteo unicinctus*), a Central and South American species; (v) a male Humboldt penguin (*Spheniscus humboldti*); (vi) a male red-crested turaco (*Tauraco erythrolophus*); (vii) a male American flamingo (*Phoenicopterus ruber*); (viii) a male domestic duck (*Anas platyrhynchos domestica*); (ix) a male emu (*Dromaius novaehollandiae*; iv to ix came from the Copenhagen Zoo); (x) adult male zebra finches from a former colony at the Duke University Medical Center (*n* = 2); (xi) adult male field-collected Anna’s hummingbirds (*Calypte anna*; *n* = 3); and (xii) 9 parrot species previously reported in Chakraborty and colleagues (2015), including budgerigars (*n* = 3 males; *n* = 2 females), peach-faced lovebirds (*n* = 3 males; *n* = 2 females), cockatiels (*n* = 3 males; *n* = 2 females), peach-fronted conure (*n* = 3 males; *n* = 2 females), a yellow lored Amazon (*n* = 1 male), a yellow crowned Amazon (*n* = 1 male), an African Grey (*n* = 1 female), a Kea (*n* = 1 female), and a blue and gold Macaw (*n* = 1 female).

For this same neural screen, wild woodpeckers from New Mexico were shot with small gauge shotguns, with brains immediately dissected out and frozen on dry ice, and the remainder of the specimens archived at the Museum of Southwestern Biology (University of New Mexico IACUC). The 6 birds (Harris hawk, Humboldt penguin, red-crested turaco, American flamingo, domestic duck, and emu) originating at the Copenhagen Zoo were anesthetized with sevoflurane and killed by an overdose of sodium barbital for reasons not related to this study (wing trauma, population management, etc.). Brains were immediately dissected out, frozen, and stored at −80°C until further processing. For sectioning, they were covered while frozen with Tissue-tek in the tissue block mold (orienting the bottom position of the telencephalon as much as possible perpendicular to the bottom surface of the mold), and the mold then placed in a dry ice ethanol bath.

Next, to examine whether specialized PV-rich brain regions were activated during drumming in downy woodpeckers, we captured additional adult birds in 3 additional experimental conditions during March to May (see below). This time period corresponds to when downy woodpeckers are actively defending territories by drumming [8–10], and thus we collected the following: (i) birds that heard playbacks of drumming but did not respond with any a drumming or aggressive vocalization (e.g., whinny call; *n* = 2); (ii) birds that heard playbacks of drumming and vocalized but did not drum (*n* = 3); and (iii) birds that drummed and vocalized in response to playback (*n* = 10). We also (iv) passively caught (*n* = 3) birds in the morning at feeding stations that were set up on known territories. For this condition, we monitored mist nets to ensure that birds were rapidly removed from the net once they were captured (<3 min). Moreover, across all conditions, we ensured birds were breeding adults based on easily observable differences in plumage between adult and juvenile animals [69]. In a subset of individuals (*n* = 5), we also verified their gonads were enlarged in a manner that suggest adulthood and reproductive activity [70].

Wild woodpeckers from North Carolina were caught in mist nets placed near established feeding stations near known breeding territories (procedure described below). The capture of these birds was approved by all federal, state, and institutional authorities (Wake Forest University IACUC). Once birds were removed from mist nets, they were immediately killed via rapid decapitation. Brains were dissected out and flash frozen in Tissue-tek embedding medium in a tissue block mold on dry ice, and then stored at −80°C until further processing. Throughout our studies, we often compared downy woodpecker brains to male zebra finch brains, which were processed just like the woodpecker brain samples under an approved Duke University IACUC protocol. Note that we never notice any differences in *PV* mRNA staining between birds killed by rapid decapitation and a bird that was killed by an overdose of isoflurane beforehand, as with the red-bellied woodpecker (*n* = 2 for rapid decapitation and *n* = 1 for isoflurane before).

### Playback experiment and behavioral recordings

We elicited territorial behavior (e.g., drumming and aggressive vocalizations [whinny calls]) by performing STIs on known downy woodpecker territories early in the morning (8:00 to 10:30 AM) prior to residents producing dawn drums or in the late afternoon during periods of quiescence (3:00 to 5:00 PM). Previous behavioral work in this species suggests that there is no difference in aggressive behavior produced during these time periods [6]. In the case of early morning behavioral trials, observers arrived on identified territories an hour before dawn and set up mist nets. Occasionally, birds were seen outside around their nest cavity. Birds caught in the morning as controls never drummed before stimulus onset.

To perform STIs, we placed a speaker (JBL; model FLIP) near territorial resident’s nest cavity as previously described [8,10]. We broadcasted the stimuli for 30 min, which consisted of drums with 15 beats with drums spaced between 4 and 50 s apart. We chose random intervals as we found it prevented the residents from habituating to the stimulus over a 30 min playback period compared to fixed intervals. These intervals correspond to the range produced when animals use drums in both spontaneous and agonistic interactions [10]. We kept the volume of each stimulus constant between trials at 80 dB, measured 1 m from the speaker (as in [10]). We selected this period of time for the STI because past work shows that the amount of vocalizing-driven gene expression is correlated with the amount of song bouts produced in a 30-min window in vocal learning birds [25,34,53]. Thus, as soon as the STI finished (after 30 min), we initiated playback at a second nearby location within the territory to capture birds in a previously set up mist net. Animals were rapidly caught [average capture time: 205.28 +/− 55.38 s (+/− 1 SEM)] and then immediately extracted from the net and killed using the same methods described above. Note that time to capture did not significantly alter IEG expression in either the dA (r^2^ = 0.01, *p* = 0.71) or the dAN (r^2^ = 0.02, *p* = 0.56).

Behavioral observations were recorded in the field using a Tascam recorder (model #HD-P2) attached to a directional microphone (Sennheiser ME66; sampling frequency = 44.1 kHz). An observer recorded the acoustic behaviors (drums and whinny calls) and flights performed (any time the bird flew over the speaker or to a new tree). For each behavior, the observers took written notes (as in [10]). Drums and whinny calls were verified and quantified after listening to audio recordings in Audacity software (v2). Among birds that drummed (*n* = 10), some birds occasionally produced whinny calls (*n* = 6). Drumming birds produced a similar number of calls compared to birds in the vocalization only conditions (*t*_6_ = 0.55, *p* = 0.60). Importantly, birds that only drummed exhibited greater *Arc* induction compared to those that drummed and vocalized (see below: Data analysis). We only captured resident birds that stayed within visual range during the entire playback period. Any animals that flew out of visual range in an experimental session were not used for this study. All field procedures were approved by the relevant Animal Care and Use Committees at Wake Forest University and Brown University.

### Synthesis of riboprobes

First, for radioactive in situ hybridization studies that localized mRNA expression of the *PV* (all species), *FoxP1* (all species), or *Lhx9* (downy woodpecker only) genes, we generated radioactive ^35^S in situ hybridization following previously described procedures [71]. To make each riboprobe, we used a clone from our zebra finch full-length cDNA collection [16,22].

Second, after identifying the initial PV specialized regions in the downy woodpecker, we made woodpecker specific probes for further experimentation. RNA from downy woodpecker brain lysate was reverse transcribed into cDNA using a previously described protocol [72]. Using this cDNA, we amplified the *PV*, *ETV1*, *RGS12*, *FoxP1*, and *Arc* (**S2 Table**). All PCR reactions contained 40 ng of cDNA, 0.5 μM of forward primer, 0.5 μM of reverse primer, Q5 polymerase (New England Biology). Reactions were then run at 98°C for 30 s, followed by 40 cycles of 98°C for 10 s; 57 to 60°C for 10 s; and 72°C for 30 s with a final extension step at 72°C for 5 min. Each reaction was then completed with a final extension step at 68°C for 5 min. Resulting PCR products were imaged on a 1% agarose gel to verify that fragments matched their expected size. We then purified the PCR products using a GeneJet PCR purification kit (Thermo Fisher) and sequenced these fragments to confirm our target amplicon (Eton Bioscience).

The resulting PCR products were inserted in into pCRII-blunt TOPO vector (Invitrogen) following the manufacturer’s instructions. These plasmids were then transformed by heat-shocking TOP10 cells for 30 s at 42°C. Next, cells were allowed to grow in SOC media for 1 h at 37°C before spread on LB agar plates containing kanamycin. The next day, individual colonies were transferred to LB liquid culture containing kanamycin. Finally, plasmids were purified from these cultures using miniprep kits (Thermo Fisher) and were then sequenced to verify the presence and orientation of the inserted PCR product.

Then, 1 μg of antisense riboprobes were synthesized using SP6 RNA polymerase (Roche) and DIG-labeling mix following the manufacturer’s instructions (Roche). Full-length *EGR1* and *Lhx9* fragments came from zebra finch cDNA cloned into PGem-TEasy vector and was synthesized using a similar protocol but with a T7 polymerase. These sequences show strong cross-hybridization with other avian species and were 91.9% and 97.47% similar, for *EGR1* and *Lhx9*, respectively. Probes were then treated with DNase for 15 min, and residual unincorporated NTPs were removed with a sodium acetate–ethanol precipitation using linear polyacrylamide as a carrier. Finally, we ran all synthesized probes on a 1% agarose gel to confirm there was a single RNA band of the appropriate size.

### Tissue processing and sectioning

Brains were sectioned in 10 or more series at 12 μm using a Leica cryostat, placed onto Colorfrost Plus microscope slides, and allowed to dry at room temperature for 30 min before being transferred in slide boxes to a −80°C freezer for long-term storage. In our initial screening for song nuclei across all 11 species, we saved all sections that were possible throughout sagittal and, in some cases, coronal planes. For the in situ hybridizations, we processed sections spaced 100 μm or more apart, depending on brain size; larger size brains allowed larger spacing between sections. For woodpeckers in the field behavior study, we focused on sections containing the PV-rich nuclei and adjacent surrounding regions.

### Radioactive in situ hybridization

We followed a previously described protocol published in detail in [71]. In brief, first, sections were fixed in 3% paraformaldehyde, rinsed in 1× phosphate buffer saline (PBS), dehydrated in an increasing ethanol series, and then air-dried. We generated radioactive ^35^S-UTP-labeled sense and antisense riboprobes by reverse transcription, and hybridized each slide containing brain sections with 1 × 10^6^ cpm at 60°C (*PV*; the lower than 65°C temperature is to obtain cross-species hybridization). We exposed the slides to NTB emulsion (Kodak, USA) diluted 1:1 in distilled water for approximately 14 to 30 d at 4°C and processed the slides with D-19 developer (Kodak) and fixer (Kodak). We visualized the bound riboprobe as silver grains, and the cell bodies by counterstaining with Cresyl violet acetate solution (Sigma, USA). Tissue incubated with the sense probe showed no significant signal above background.

### Colormetric in situ hybridization

Brain sections were first fixed in 4% paraformaldehyde for 5 min and then washed in PBS twice for 3 min each. Sections were then acetylated for 10 min in a 0.1-M TEA solution with 0.33% acetic anhydride, rinsed once with PBS, and then dehydrated in 70%, 95%, and 100% ethanol. Sections were next incubated in prehybridization buffer (5× SSC, 1× Denhardt’s, 250 μg/mL tRNA, 500 μg/mL Herring sperm DNA, 50% formamide in DI water) at room temperature for 1 h. Slides were then transferred to hybridization buffer (300 mM NaCl, 20 mM Tris-HCl (pH 8), 5 mM EDTA, 10 mM Na_2_HPO_4_ (pH = 7.2), 10% dextran sulfate, 1× Denhardt’s, 500 μg/mL tRNA, 200 μg/mL Herring sperm DNA, 50% formamide in DI water) with the DIG-labeled riboprobe. Slides were cover slipped, sealed by immersion in mineral oil, and incubated overnight at 63 to 65°C. On the following day, sections were rinsed and de-coverslipped in chloroform, washed in 5× SSC for 10 min, and then washed in 0.2× SSC 4 times for 30 min. Sections were then rinsed briefly 100 mM Tris (pH 7.5); 150 mM NaCl, and blocked for 60 min at RT in blocking buffer (0.1 M Tris, 150 mM NaCl, 20% normal sheep serum). Slides were then transferred to blocking buffer with an alkaline phosphatase conjugated anti-DIG antibody (Roche) an allowed to incubate at 4°C overnight. Then, the slides were washed 3 × 10 min in 100 mM Tris (pH 7.5); 150 mM NaCl and then equilibrated in 100 mM Tris (pH 9.5) for 10 min before transferring slides to the detection solution containing the alkaline phosphatase substrates Nitro-Blue Tetrazolium Chloride (NBT) and 5-Bromo-4-Chloro-3-Indolyl-phosphate p-Toluidine Salt (BCIP) (Vector Labs) diluted in 100 mM Tris (pH 9.5) according to the manufacturer’s instructions. After sections were sufficiently developed, they were washed twice in 1× PBS for 3 min and then briefly rinsed in DI water before being counterstained in Nuclear FastRed (diluted 1:4 in DI water; Vector Labs). Finally, sections were rinsed in DI water to remove excess counterstain and dehydrated in 100% ethanol. Slides were then mounted with VectaMount (Vector labs) and cover slipped.

### Fluorescent in situ hybridization

Brain sections were first fixed in 4% paraformaldehyde for 5 min and then washed in PBS twice for 3 min each. Sections were then acetylated for 10 min in a 0.1-M TEA solution with 0.33% acetic anhydride, rinsed once with PBS, and then serially dehydrated in 70%, 95%, and 100% ethanol. Sections were next incubated in hybridization at room temperature for 1 h. Slides were then transferred to hybridization buffer with antisense DIG (*PV* or *FoxP1* mRNA)-labeled riboprobe. Slides were cover slipped, sealed, and incubated overnight at 60°C. The following day, sections were rinsed, and cover slips removed, washed in 5× SSC for 10 min, and then washed in 0.2× SSC 3 times for 20 min. After rinsing slides in TNT (Tris-NaCl-Tween) buffer, slides were then incubated in blocking buffer (0.5% blocking reagent [Roche] diluted in TNT buffer) for 1 h at room temperature. Next, slides were incubated in anti-DIG-HRP antibody diluted in TNT buffer (1:2,000) overnight at 4°C. The following day, slides were washed 3 times in TNT buffer for 10 min each. We then incubated slides in FITC-TSA (1:150) diluted in amplification buffer (Akoya Biosciences) for 10 min. Finally, slides were washed 3 times in PBS and then mounted in Prolong Gold with DAPI.

### Image acquisition and quantification

Colormetric in situ slides were visualized on a Ziess Axiozoom V16 stereoscope. All images for IEG quantification were taken at 32× optical zoom using ZenBlue. The number of *PV*+ in the dA, dAN, and the regions forebrain that surround these nuclei (intermediate arcopallium (AI) and anterior nidopallium (AN), respectively). For the AI, we counted all the cells within a fixed region (500 × 500 μm square) that was directly below the *PV*-rich dorsal arcopallial region (see **Fig 7** for more details). To count cells in AN, we restricted our counts to the region that immediately surrounded dAN (see **Fig 7** for more details). For all 4 regions, PV cells counts were normalized to area in which cells were counted (number of PV+ cells/μm^2^).

To quantify both *EGR1* and *Arc*, regions of interests were determined by aligning an adjacent PV image in Photoshop and then outlining the region of PV-rich cells. Two investigators who were blind to the condition of the birds counted the total number of *Arc* or *EGR1* expressing cells in these regions of interest. The region we chose is consistent with detailed reports annotating the AI [27]. For the anterior mesopallium (AM), we used previously defined boundaries as in Feenders and colleagues [3]. Finally, we determined the boundaries for each auditory region by using brain atlases [29] and IEG studies from passerine songbirds, parrots, and hummingbirds [25,26]. Cell counts were first normalized to the area (μm^2^) of each brain region. Then, as in many previous analyses with IEG analyses in the song system [15,32,43], we normalized expression of all animals captures during playback experiments to the average expression of passive (no playback) controls. Accordingly, this generated normalized *Arc* and *EGR1* mRNA expression. Fluorescent in situ hybridization for *PV* mRNA were visualized using a Zeiss LSM 710 confocal microscope using a 10× objective. Tile scans of entire sections were acquired in the Zen software.

### Data analysis

We performed analyses in R, after log (1+x) transforming normalized *Arc* cell counts to achieve normality. Using Q–Q plots and Shapiro-Wilk tests, we verified that these transformations did in fact yield a more normally distributed data. We used a one-way analysis of variance (ANOVA) to investigate whether *Arc* and *EGR1* expression differed in the arcopallial regions (dA and AI), nidopallium (dAN and AN), and auditory regions (NCM and CMM) between drumming, vocalizing, silent, and passively behaving woodpeckers. For these analyses, the drumming condition consisted of birds that only drummed (*n* = 4) and those that drummed and produced a small number of whinny calls (*n* = 6). Since *Arc* expression levels did not differ between these 2 groups for either the dA (*t*_8_ = 0.46, *p* = 0.65) or the dAN (*t*_8_ = 0.12, *p* = 0.91), we collapsed these into a single condition called “drumming”. Linear regression analyses were performed to determine if *Arc* mRNA expression in the arcopallial or nidopallial regions were associated with the number of drums, vocalizations (whinny calls), aggressive flights, or total behavior (drums, vocalizations, and flights) produced during the STI. To investigate *Arc* “contrast” in putative drum nuclei (dA and dAN), we took the ratio of normalized *Arc* cell counts (Arc+ cells/μm^2^) in dA or dAN to normalized cell counts in in AI or AN (e.g., dA:AI or dAN:AN). To evaluate whether drumming birds had higher contrast relative to nondrumming birds, we performed an independent sample *t* test for each ratio.

## Supporting information

S1 Fig Lack of forebrain structures identified with *parvalbumin* (*PV*) mRNA expression in penguin and emu. (A-B) Representative radioactive in situ hybridization microscope images of *PV* mRNA in species of (A) Humbolt penguin (*Spheniscus humboldti*) and (B) emu (*Dromaius novaehollandia*). In contrast to hummingbirds and woodpeckers (see Fig 2E and 2F), *PV*-rich forebrain nuclei were absent in these 2 species. Each scale bar is equal to 2 mm. Neuroanatomical markers shown in (A) are as follows: Hyper, hyperpallium; Meso, mesopallium; Nido, nidopallium; GP, globus pallidus; T, Thalamus; Ot, optic tectum; St, striatum; Arco, arcopallium. *Photo credits*: *penguin from Mariana Ruiz Villarreal (CC BY 2*.*0) and emu from Daderot (CC Public Domain via wikimedia)*.
(TIFF)

S2 Fig Coronal plane of the forebrain structures in male budgerigars (parrot) and downy woodpecker illustrating specialized *parvalbumin* (*PV*) mRNA expression, with such expression patterns absent in a Harris hawk. (A) Representative radioactive in situ hybridization of microscope images of *PV* mRNA, in budgerigar (parrot) pallial song nuclei (NAO core, AAC core and shell, and NLC core and shell). Sections modified from Chakraborty and colleagues (2015) with permission from Dr. Jarvis, who is also an author on the current paper. (B) Coronal sections of the woodpecker brain showing the analogous locations for dAN in the anterior nidopallium and dNA in the arcopallium. (C) Comparable coronal sections in a Harris hawk that show PV expression in many positive control areas (see S1 Table). However, unlike the parrot and woodpecker, there was no specialized expression in the arco- or nidopallium. The in situs have cresyl violet as a counter stain.
(TIFF)

S3 Fig Parvalbumin (PV) specialization in the male and female downy woodpecker arcopallium and nidopallium. (A-D) Representative *PV* mRNA expression (green) from fluorescent in situ hybridization experiments at low-magnification (tile scan) and (Ai and Ci) high-magnification illustrations of neuroanatomical regions with *PV* up-regulation in the male downy woodpecker brain. (E and F) Representative *PV* mRNA staining in the DLN, dAN, and dA of a female downy woodpecker. Blue signal is a DAPI nuclear stain. All scale bars are 1 mm. Asterisks (*) indicate folds on tissue. *Photo credits*: *male downy woodpecker from Greg Schechter*, *and female downy woodpecker from Ken Thomas (CC Public Domain via WikiMedia)*.
(TIF)

S4 Fig Parvalbumin (PV) specialization through the downy woodpecker arcopallium. (A) Colormetric and (B) radioactive in situ hybridization illustrating specialized patterns of *PV* or (C) arcopallium-enriched *Lim homeobox 9 (Lhx9)* mRNA expression. Medial parasagittal sections through the downy woodpecker arcopallium show that *PV* has specialized expression in dA of the dorsal and intermediate arcopallium. These findings are consistent with the 2 different types of probe labeling and hybridization methods. Although *Lhx9* demarcates most of the woodpecker arcopallium, it is largely absent in the anterior arcopallium, as seen in songbirds [18]. Dashed lines indicate the arcopallial boundary. Scale bars represent 500 μm.
(TIFF)

S5 Fig Assessing striatal specializations and markers for the woodpecker arcopallium. Two markers, (A) *ETV1* and (B) *Lhx9* (radioactive in situ hybridization), were used to delineate the boundary of the arcopallium and nidopallium. (C-F) Representative in situ hybridization images (inverted black and white colormetric) of (C and D) *FoxP1* and (E and F) *RGS12* in zebra finch and downy woodpecker. Both genes are significantly enriched in the zebra finch Area X (striatal nucleus); however, neither demarcates a specialized region within the woodpecker striatum. *FoxP1* allows for the clear delineation of all nidopallial-striatal boundaries. Both reveal similar patterns to zebra finches (see [23]). Data from *Lhx9* was collected through radioactive in situ hybridization (see Materials and methods for details). Hyp: hyperpallium; arco: arcopallium; nido: nidopallium; Meso: mesopallium. Scale bar, 500 μm.
(TIF)

S6 Fig Behaviorally induced changes in *EGR1* and *Arc* expression in the arcopallium and nidopallium of male downy woodpeckers. In situ hybridization microscope images of *EGR1* on adjacent *parvalbumin* (*PV*) sections in the (A, C, E) dorsal arcopallial (dA) and (G, I, K) drumming nucleus of the anterior nidopallium (dAN) or of male downy woodpeckers that were passively caught, low drummers (individual represented in C,D,I, and J produced 5 drums) and high drummers (individual represented in E,F,K, and L produced 49 drums) during simulated territorial intrusions (STIs). In situ hybridization microscope images of *Arc* on adjacent *parvalbumin* (*PV*) sections in the (B, D, F) dorsal arcopallial (dA) and (H, J, L) drumming nucleus of the anterior nidopallium (dAN) or of male downy woodpeckers that were passively caught, low drummers and high drummers during STIs. (M, N) Violin plots (horizontal line denotes median) of differences in *EGR1* gene expression in the *PV*-rich (M) dA or (N) dAN nuclei, respectively, of male downy woodpeckers caught after producing different behaviors. *EGR1* mRNA expression significantly differed in the dA (*F*_*3*,*14*_ = 3.98, *p* = 0.03), but we did not detect any differences *EGR1* in the dNA across behavioral conditions (*F*_*3*,*14*_ = 0.32, *p* = 0.81). Data for M and N can be found in S2 Data.
(TIF)

S7 Fig Representative examples of drums during playback. (A-B) Each recording includes an example of a stimulus drum (orange rectangles) being broadcast over a speaker and a resident (red rectangles) responding to this stimulus by producing a drum.
(TIFF)

S8 Fig Graphs showing no relationship between Arc mRNA expression and capture time in the dA or dAN. Data for these analyses can be found in S2 Data.
(TIFF)

S1 Table *Parvalbumin* (*PV*) mRNA staining with radioactive in situ hybridization across species (Anna’s hummingbird, *Calypte anna*; Downy woodpecker, *Dryobates pubescens*; Harris hawk, *Parabuteo unicinctus*; American flamingo, *Phoenicopterus ruber*; Red-rested turaco, *Tauraco erythrolophus*; Domestic duck, *Anas platyrhynchos domesticus*; Emu, *Dromaius novaehollandiae*; Humbolt penguin, *Spheniscus humboldti*). *PV* expression present in a brain area is denoted by a plus (+) sign; PV absent in a brain area is denoted by a minus (–) sign.
(DOCX)

S2 Table Primers used to generate downy woodpecker in situ probes. (DOCX)

S1 Data Parvalbumin cell count data. (CSV)

S2 Data Behavioral and normalized immediate early gene (ZENK and Arc) expression. (CSV)
