# Supplementary figures and images for: Forebrain nuclei linked to woodpecker territorial drum displays mirror those that enable vocal learning in songbirds
Source: PLoS Biol. 2022 Sep 20;20(9):e3001751. doi: 10.1371/journal.pbio.3001751 (PMC9488818; doi:10.1371/journal.pbio.3001751)

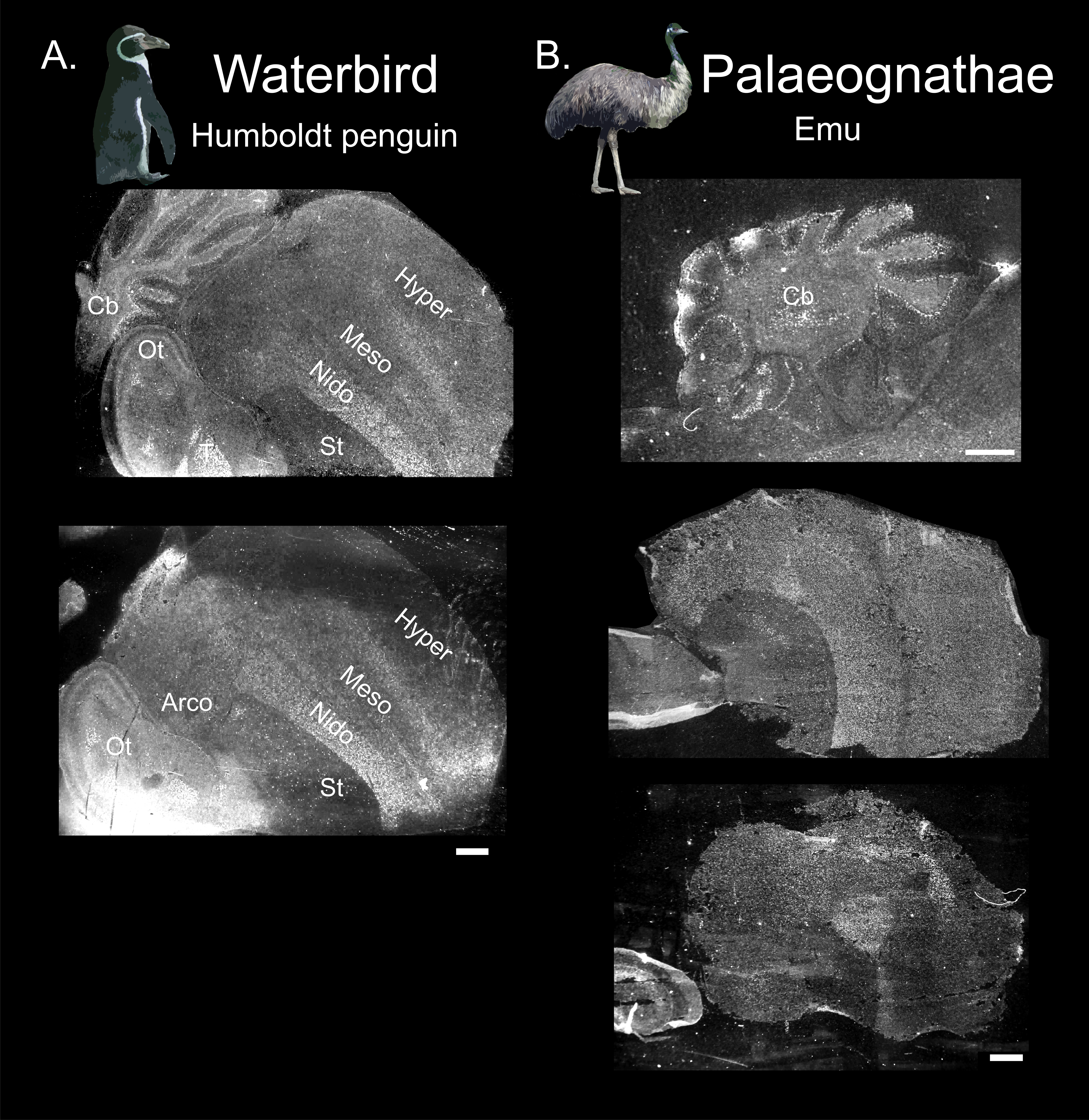

Supplement: S1 Fig — (A-B) Representative radioactive in situ hybridization microscope images of PV mRNA in species of (A) Humbolt penguin (Spheniscus humboldti) and (B) emu (Dromaius novaehollandia). In contrast to hummingbirds and woodpeckers (see Fig 2E and 2F), PV-rich forebrain nuclei were absent in these 2 species. Each scale bar is equal to 2 mm. Neuroanatomical markers shown in (A) are as follows: Hyper, hyperpallium; Meso, mesopallium; Nido, nidopallium; GP, globus pallidus; T, Thalamus; Ot, optic tectum; St, striatum; Arco, arcopallium. Photo credits: penguin from Mariana Ruiz Villarreal (CC BY 2.0) and emu from Daderot (CC Public Domain via wikimedia). (TIFF) [file pbio.3001751.s001.tiff]

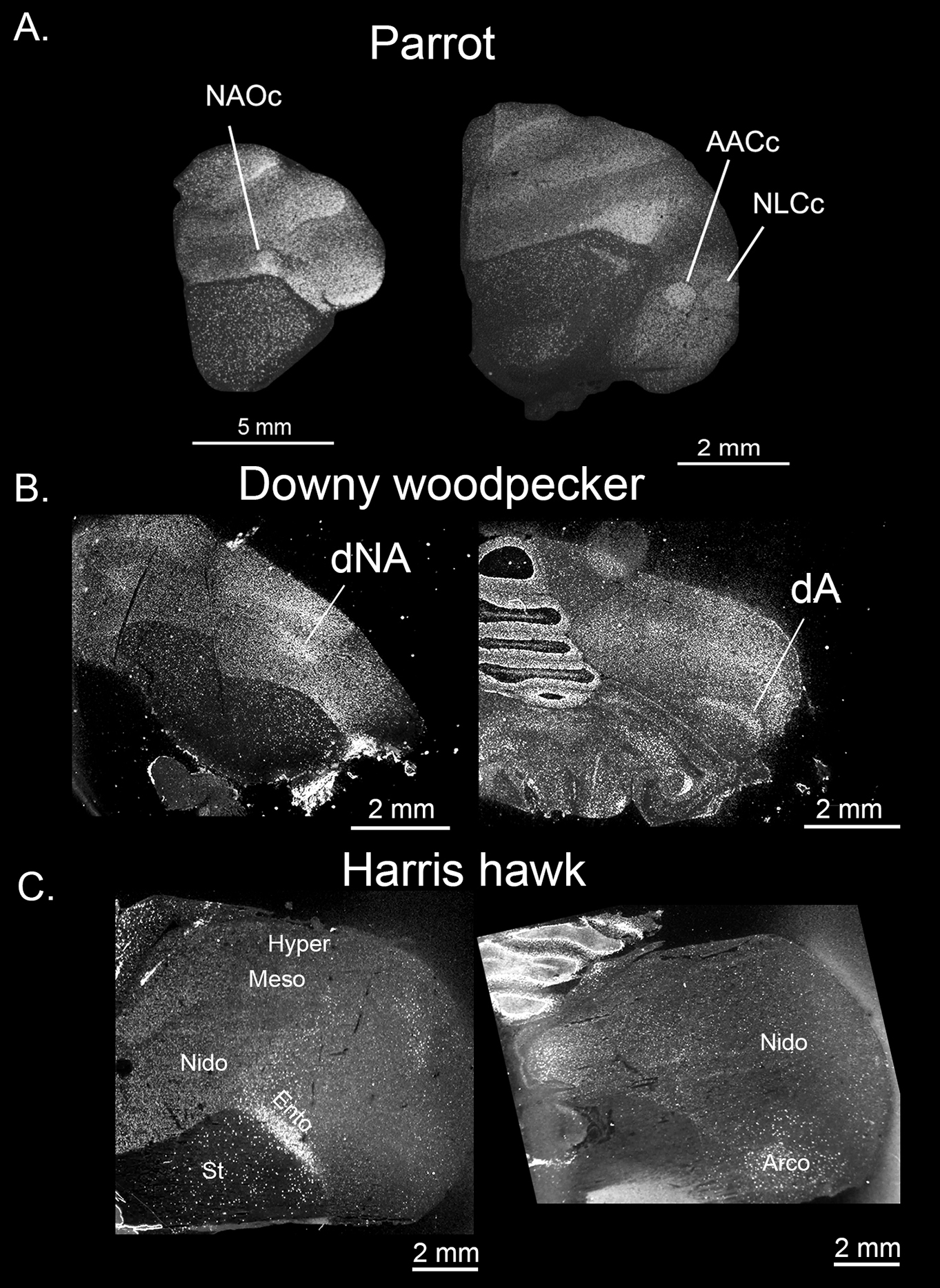

Supplement: S2 Fig — (A) Representative radioactive in situ hybridization of microscope images of PV mRNA, in budgerigar (parrot) pallial song nuclei (NAO core, AAC core and shell, and NLC core and shell). Sections modified from Chakraborty and colleagues (2015) with permission from Dr. Jarvis, who is also an author on the current paper. (B) Coronal sections of the woodpecker brain showing the analogous locations for dAN in the anterior nidopallium and dNA in the arcopallium. (C) Comparable coronal sections in a Harris hawk that show PV expression in many positive control areas (see S1 Table). However, unlike the parrot and woodpecker, there was no specialized expression in the arco- or nidopallium. The in situs have cresyl violet as a counter stain. (TIFF) [file pbio.3001751.s002.tiff]

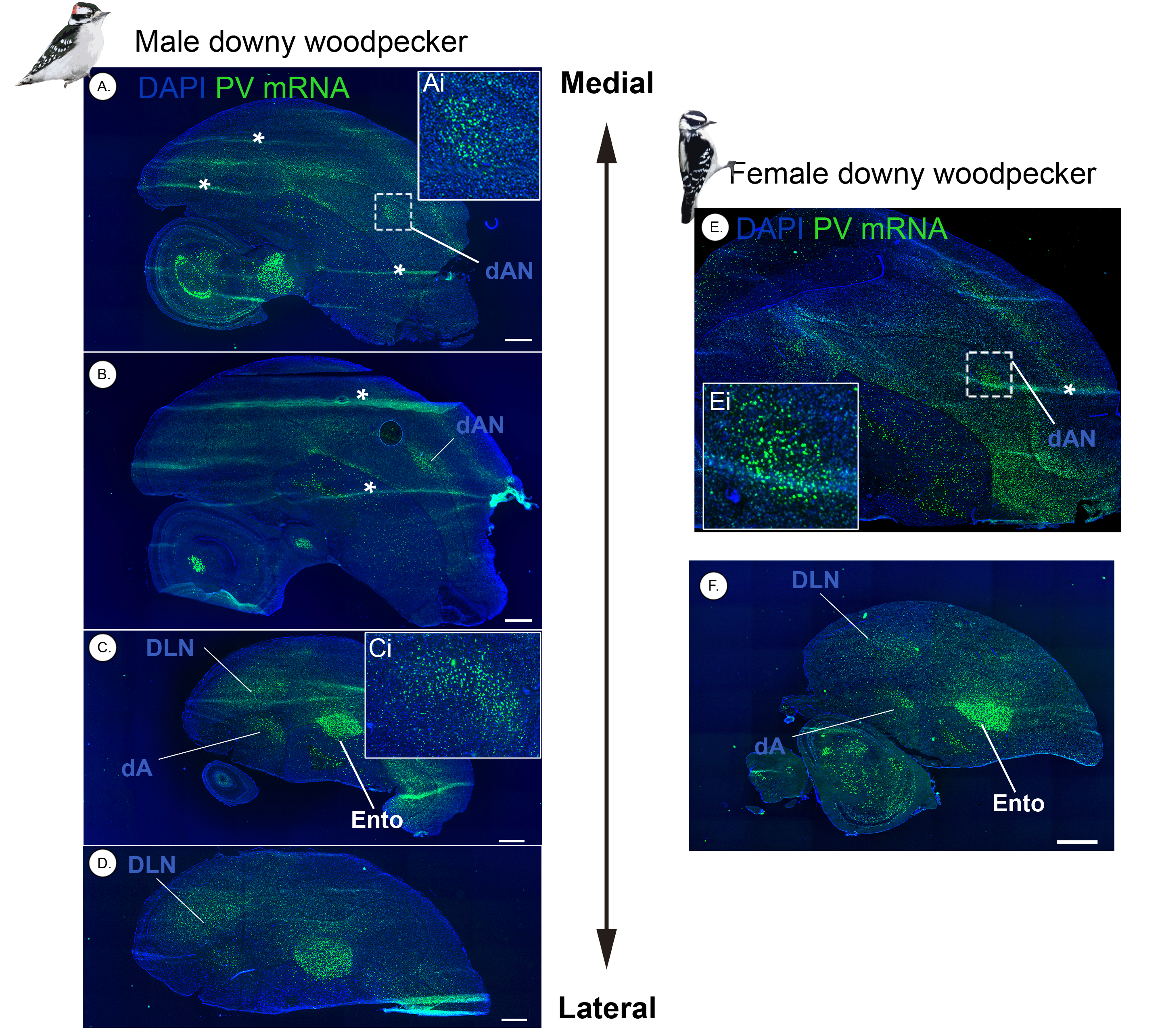

Supplement: S3 Fig — (A-D) Representative PV mRNA expression (green) from fluorescent in situ hybridization experiments at low-magnification (tile scan) and (Ai and Ci) high-magnification illustrations of neuroanatomical regions with PV up-regulation in the male downy woodpecker brain. (E and F) Representative PV mRNA staining in the DLN, dAN, and dA of a female downy woodpecker. Blue signal is a DAPI nuclear stain. All scale bars are 1 mm. Asterisks (*) indicate folds on tissue. Photo credits: male downy woodpecker from Greg Schechter, and female downy woodpecker from Ken Thomas (CC Public Domain via WikiMedia). (TIF) [file pbio.3001751.s003.tif]

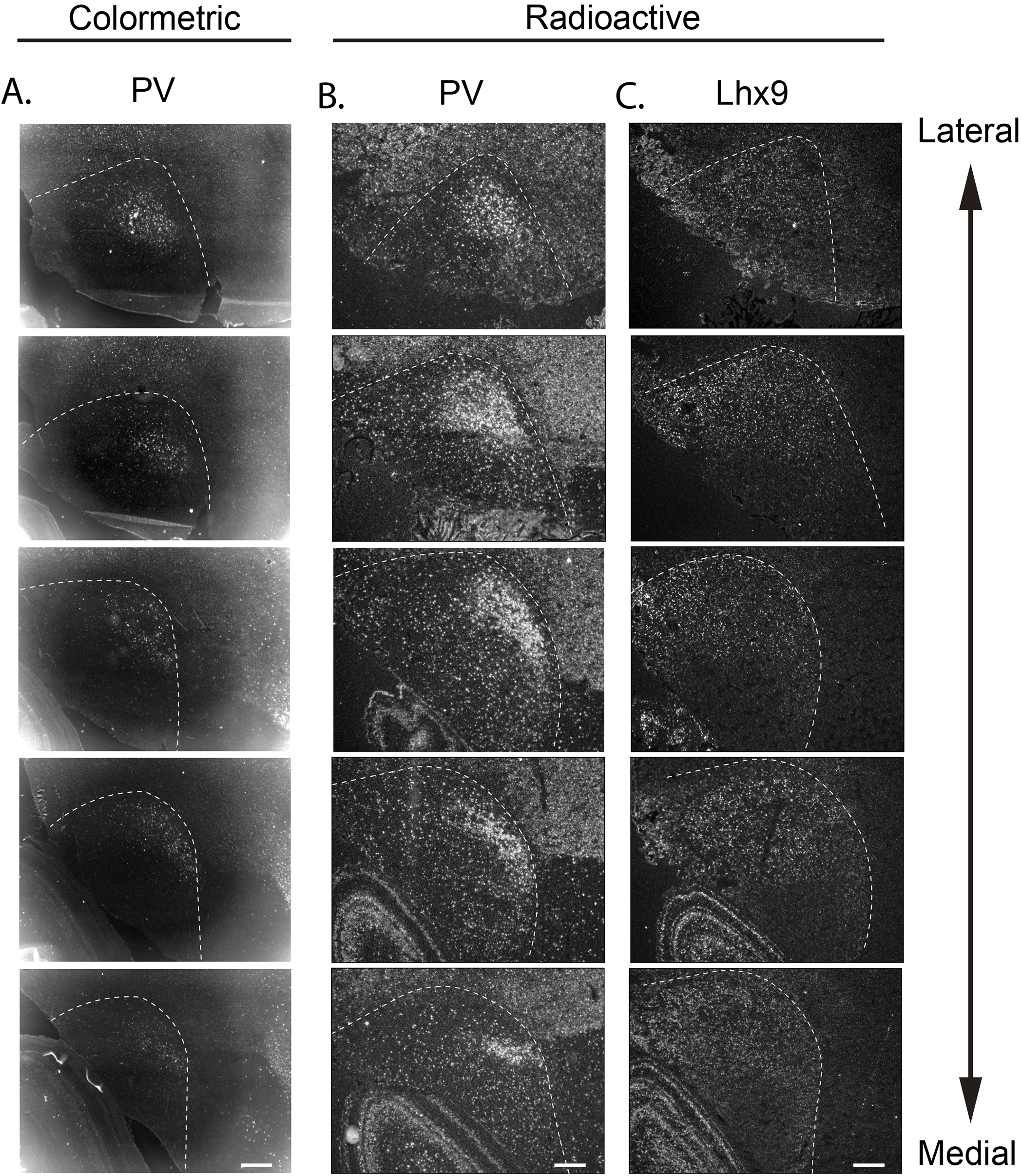

Supplement: S4 Fig — (A) Colormetric and (B) radioactive in situ hybridization illustrating specialized patterns of PV or (C) arcopallium-enriched Lim homeobox 9 (Lhx9) mRNA expression. Medial parasagittal sections through the downy woodpecker arcopallium show that PV has specialized expression in dA of the dorsal and intermediate arcopallium. These findings are consistent with the 2 different types of probe labeling and hybridization methods. Although Lhx9 demarcates most of the woodpecker arcopallium, it is largely absent in the anterior arcopallium, as seen in songbirds [18]. Dashed lines indicate the arcopallial boundary. Scale bars represent 500 μm. (TIFF) [file pbio.3001751.s004.tiff]

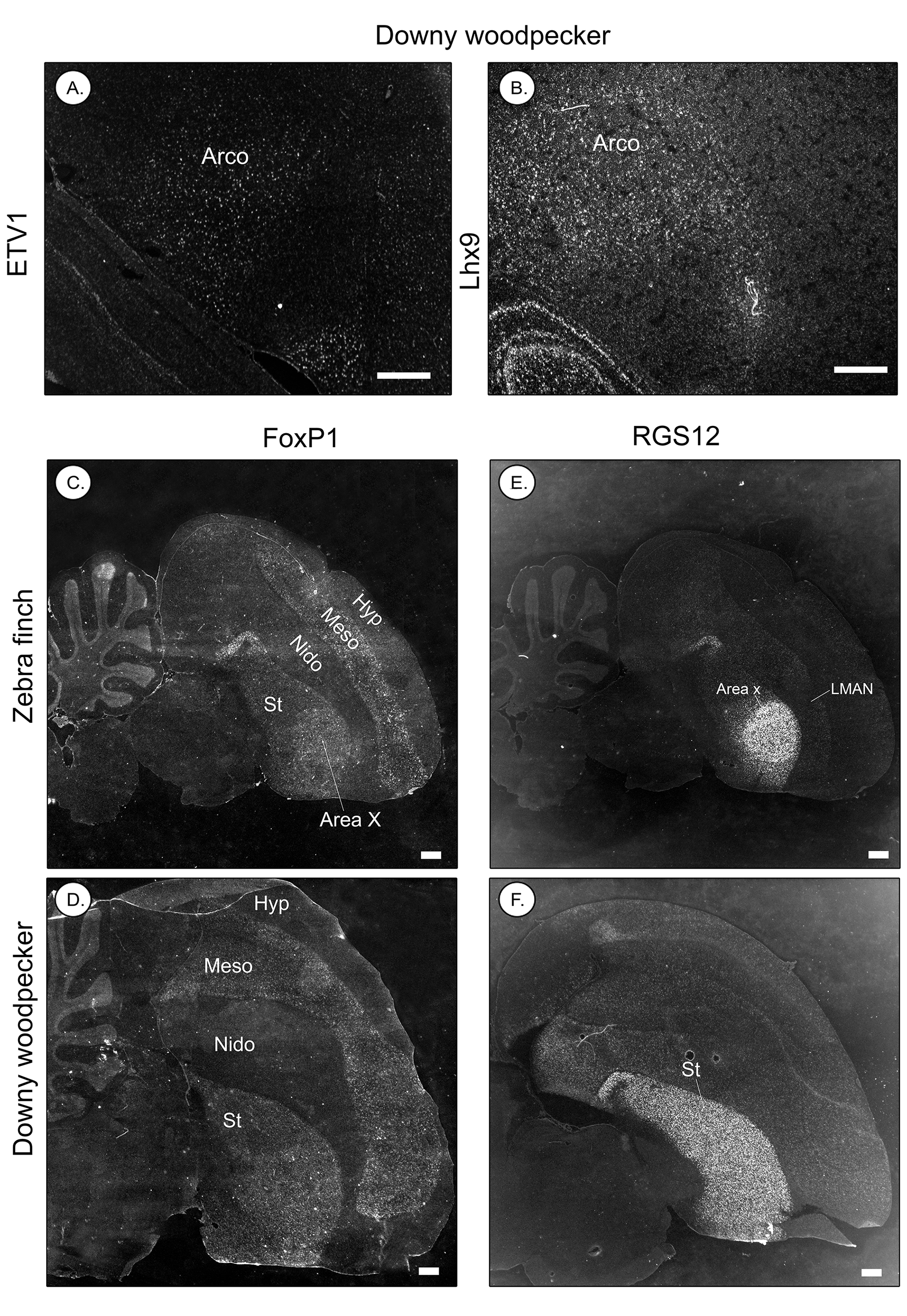

Supplement: S5 Fig — Two markers, (A) ETV1 and (B) Lhx9 (radioactive in situ hybridization), were used to delineate the boundary of the arcopallium and nidopallium. (C-F) Representative in situ hybridization images (inverted black and white colormetric) of (C and D) FoxP1 and (E and F) RGS12 in zebra finch and downy woodpecker. Both genes are significantly enriched in the zebra finch Area X (striatal nucleus); however, neither demarcates a specialized region within the woodpecker striatum. FoxP1 allows for the clear delineation of all nidopallial-striatal boundaries. Both reveal similar patterns to zebra finches (see [23]). Data from Lhx9 was collected through radioactive in situ hybridization (see Materials and methods for details). Hyp: hyperpallium; arco: arcopallium; nido: nidopallium; Meso: mesopallium. Scale bar, 500 μm. (TIF) [file pbio.3001751.s005.tif]

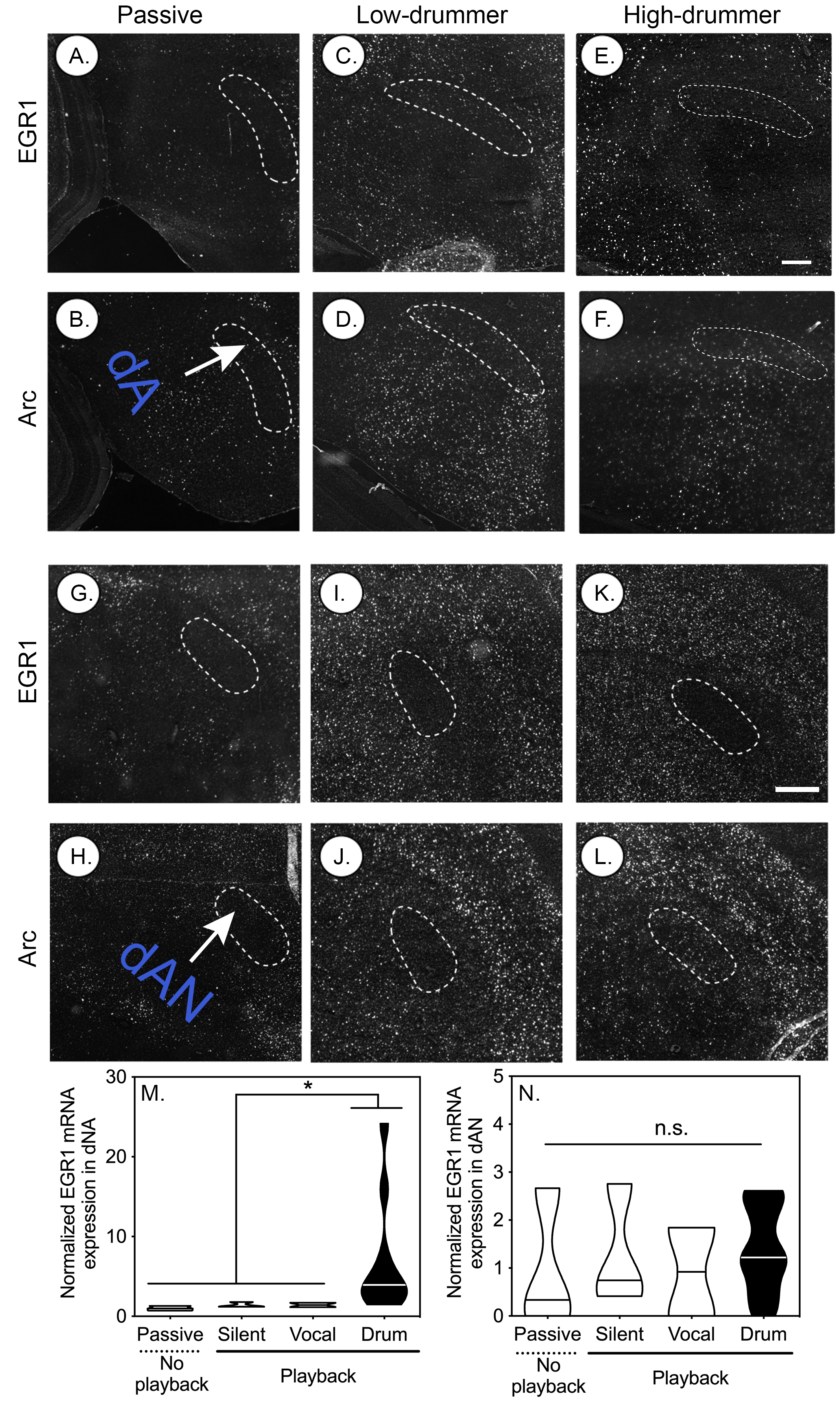

Supplement: S6 Fig — In situ hybridization microscope images of EGR1 on adjacent parvalbumin (PV) sections in the (A, C, E) dorsal arcopallial (dA) and (G, I, K) drumming nucleus of the anterior nidopallium (dAN) or of male downy woodpeckers that were passively caught, low drummers (individual represented in C,D,I, and J produced 5 drums) and high drummers (individual represented in E,F,K, and L produced 49 drums) during simulated territorial intrusions (STIs). In situ hybridization microscope images of Arc on adjacent parvalbumin (PV) sections in the (B, D, F) dorsal arcopallial (dA) and (H, J, L) drumming nucleus of the anterior nidopallium (dAN) or of male downy woodpeckers that were passively caught, low drummers and high drummers during STIs. (M, N) Violin plots (horizontal line denotes median) of differences in EGR1 gene expression in the PV-rich (M) dA or (N) dAN nuclei, respectively, of male downy woodpeckers caught after producing different behaviors. EGR1 mRNA expression significantly differed in the dA (F3,14 = 3.98, p = 0.03), but we did not detect any differences EGR1 in the dNA across behavioral conditions (F3,14 = 0.32, p = 0.81). Data for M and N can be found in S2 Data. (TIF) [file pbio.3001751.s006.tif]

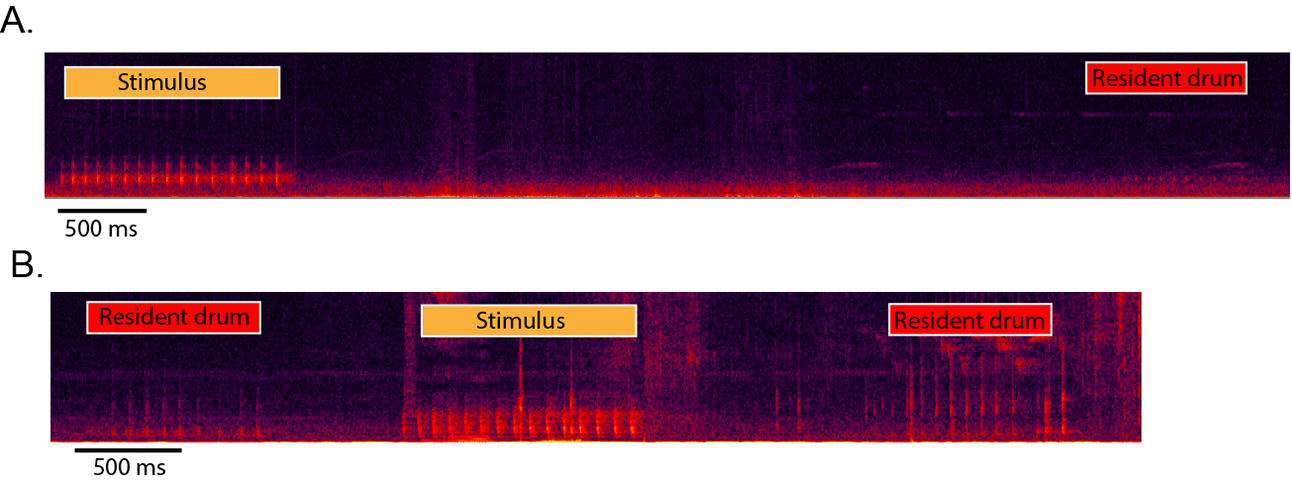

Supplement: S7 Fig — (A-B) Each recording includes an example of a stimulus drum (orange rectangles) being broadcast over a speaker and a resident (red rectangles) responding to this stimulus by producing a drum. (TIFF) [file pbio.3001751.s007.tiff]

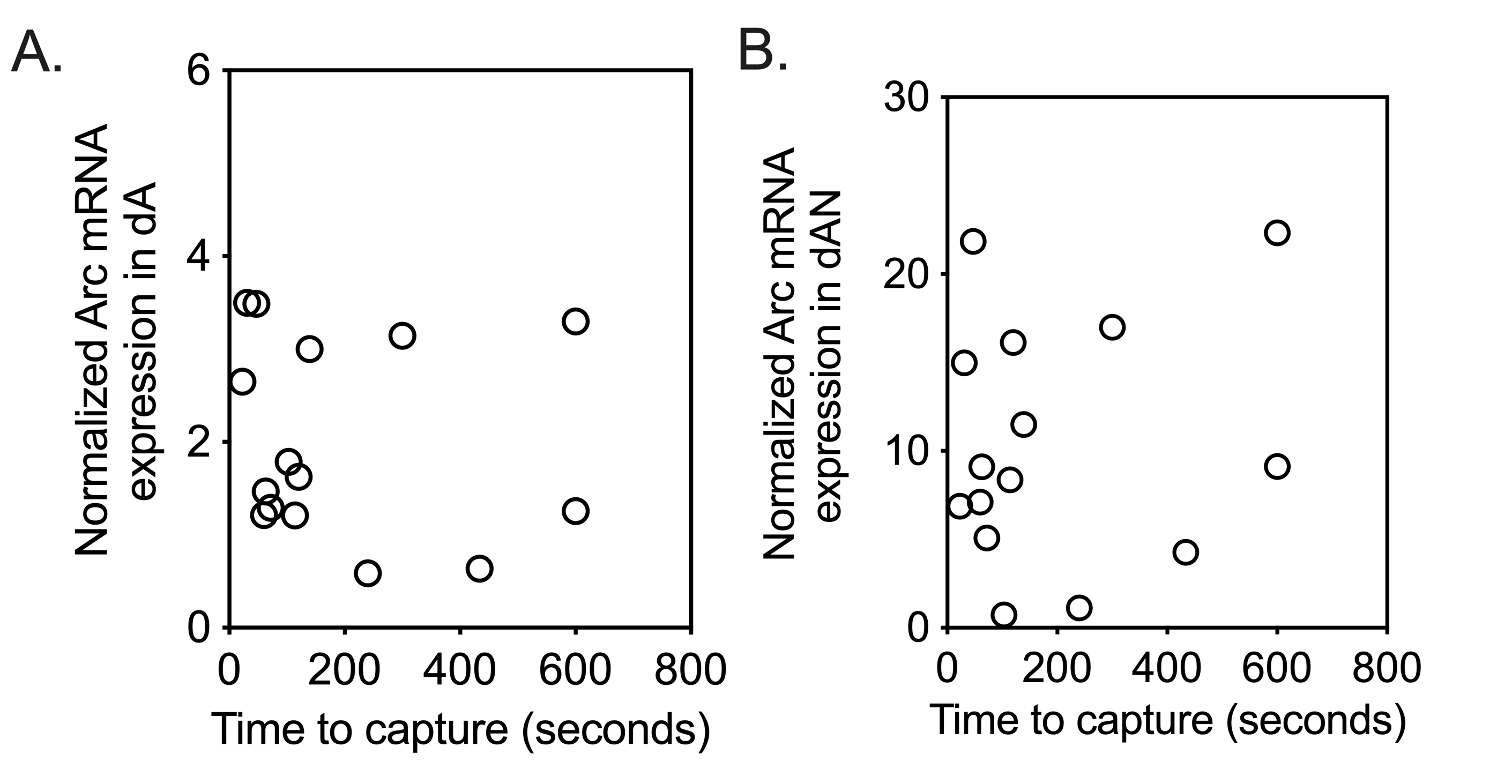

Supplement: S8 Fig — Data for these analyses can be found in S2 Data. (TIFF) [file pbio.3001751.s008.tiff]
