# Supplementary material for: Forebrain nuclei linked to woodpecker territorial drum displays mirror those that enable vocal learning in songbirds
Source: PLoS Biol. 2022 Sep 20;20(9):e3001751. doi: 10.1371/journal.pbio.3001751 (PMC9488818; doi:10.1371/journal.pbio.3001751)
Supplement: S1 Table — PV expression present in a brain area is denoted by a plus (+) sign; PV absent in a brain area is denoted by a minus (–) sign. (DOCX) [file pbio.3001751.s009.docx]

**Table S1**. ***Parvalbumin* (*PV*) mRNA staining with radioactive *in situ* hybridization across species (Anna’s hummingbird, *Calypte anna*; Downy woodpecker, *Dryobates pubescens*; Harris hawk, *Parabuteo unicinctus*; American flamingo, *Phoenicopterus ruber*; Red-rested turaco, *Tauraco erythrolophus*; Domestic duck, *Anas platyrhynchos domesticus*; Emu, *Dromaius novaehollandiae*; Humbolt penguin, *Spheniscus humboldti*. *PV* expression present in a brain area is denoted by a plus (+) sign; PV absent in a brain area is denoted by a minus (-) sign.**

|  | **Anna’s hummingbird (vocal learner)** | **Downy woodpecker** | **Harris hawk** | **American flamingo** | **Red-crested turaco** | **Domestic duck** | **Emu** | **Humboldt penguin** |
| --- | --- | --- | --- | --- | --- | --- | --- | --- |
| Entopallium | + | + | + | + | + | + | + | + |
| Globus pallidus | + | + | + | + | + | + | + | + |
| Nucleus rotundus | + | + | + | + | + | + | + | + |
| MLD | + | + | + | + | + | + | + | + |
| Subpretectal nucleus | + | + | + | + | + | + | + | + |
| Purkinje cells | + | + | + | + | + | + | + | + |
| Cerebellar nucleus | + | + | + | + | + | + | + | + |
| PV-rich nucleus in the anterior nidopallium | + (VAN) | + (DAN) | - | - | - | - | - | - |
| PV-rich nucleus in the lateral nidopallium | + (VLN) | + (DLN) | - | - | - | - | - | - |
| PV-rich nucleus in the arcopallium | + (VA) | + (dNA) | - | - | - | - | - | - |
