# Supplementary material for: Forebrain nuclei linked to woodpecker territorial drum displays mirror those that enable vocal learning in songbirds
Source: PLoS Biol. 2022 Sep 20;20(9):e3001751. doi: 10.1371/journal.pbio.3001751 (PMC9488818; doi:10.1371/journal.pbio.3001751)
Supplement: S2 Table — (DOCX) [file pbio.3001751.s010.docx]

**Table S2.** **Primers used to generate downy woodpecker in situ probes**

| **Target** | **Forward primer** | **Reverse primer** |
| --- | --- | --- |
| *Parvalbumin* | AGTTGCAGGATGGCTATGACT | GCACAATTACAGAGTTGCAGGA |
| *ETV1* | TTCAGAAGTGCCTAGCTGCC | CATTGATGTGGCGCTCCATG |
| *FoxP1* | AGCAACTCCAAGTCCTGCTC | CTTGCAGGCGCTCTTTATCT |
| *RGS12* | TGAGCCCTCAAGATTGCCTG | TCTTTGACAGCCGGACTTCC |
| *Arc* | TTGCCAACAAGCCCAATGTG | CGGGGATCCTCAAAGACCTG |
